# Supplementary figures and images for: Interleukin-1 prevents SARS-CoV-2-induced membrane fusion to restrict viral transmission via induction of actin bundles (part 1 of 2)
Source: eLife. 2025 Feb 12;13:RP98593. doi: 10.7554/eLife.98593 (PMC11820142; doi:10.7554/eLife.98593)

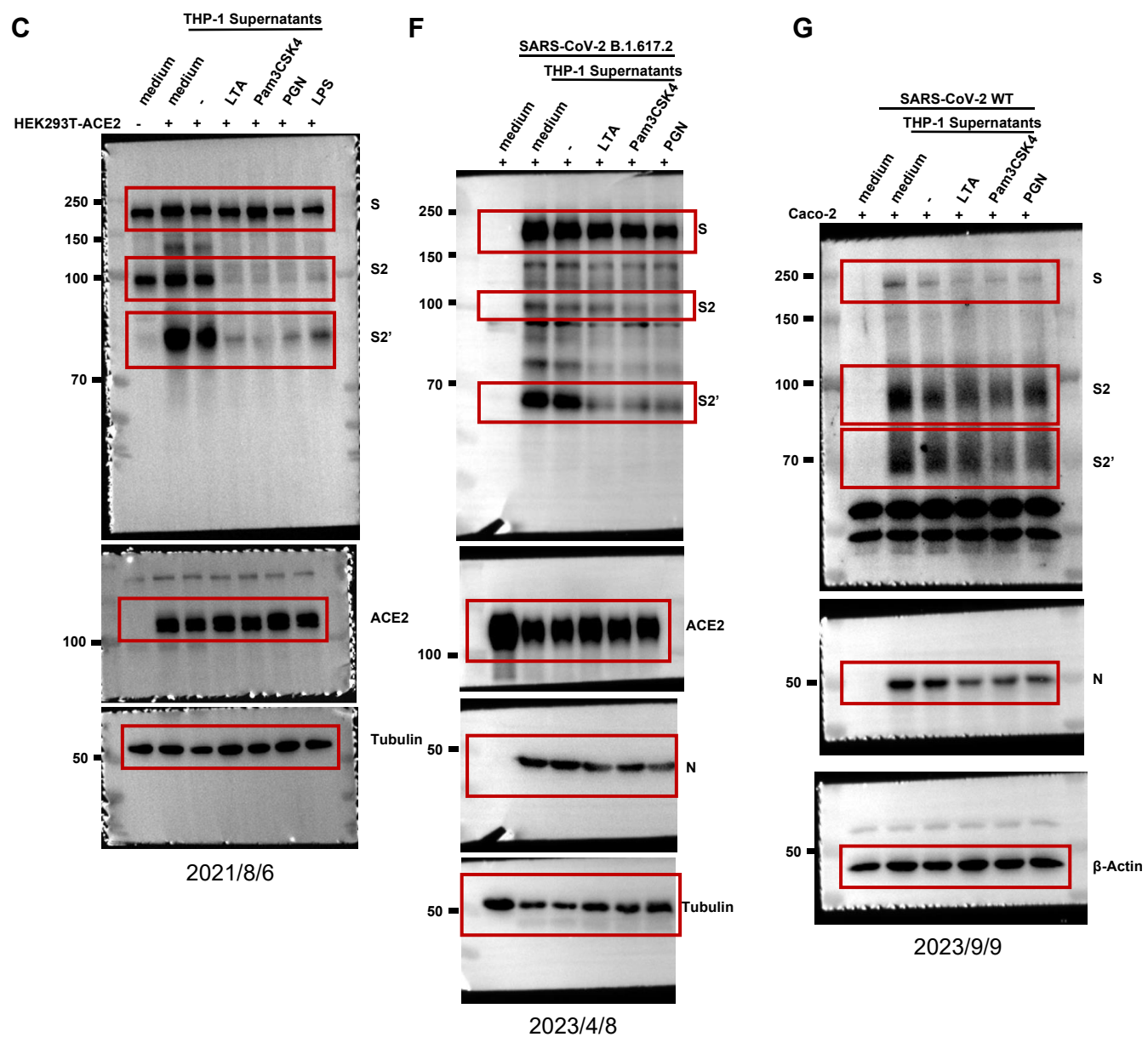

**Figure 1-Source Data 1.** Original membranes corresponding to Figure 1C, Figure 1F and Figure 1G.

Supplement: Figure 1—source data 1. [file elife-98593-fig1-data1.pdf]

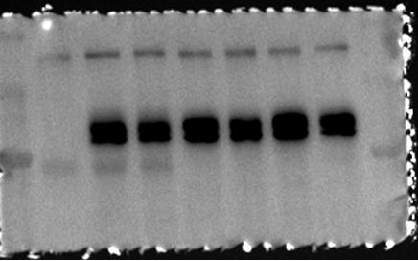

Supplement: Figure 1—source data 2. [file elife-98593-fig1-data2.zip › Figure 1 - Source data 2/Figure 1C-ACE2.tif]

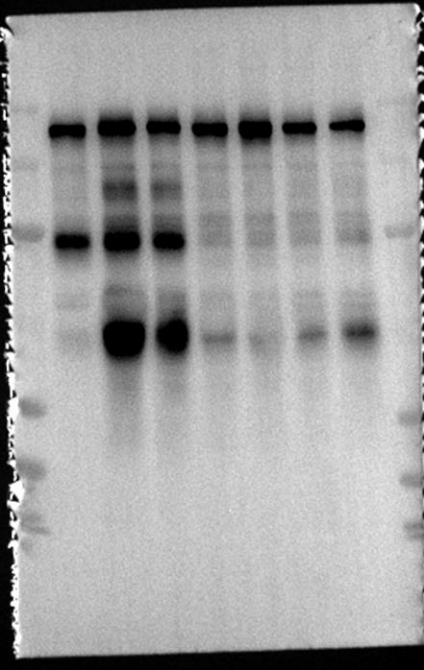

Supplement: Figure 1—source data 2. [file elife-98593-fig1-data2.zip › Figure 1 - Source data 2/Figure 1C-S.tif]

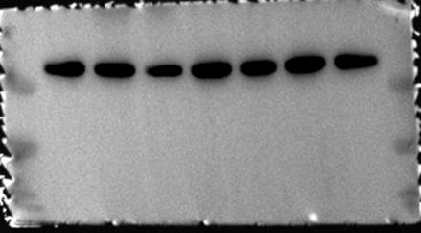

Supplement: Figure 1—source data 2. [file elife-98593-fig1-data2.zip › Figure 1 - Source data 2/Figure 1C-Tubulin.tif]

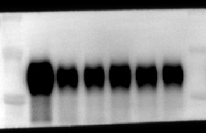

Supplement: Figure 1—source data 2. [file elife-98593-fig1-data2.zip › Figure 1 - Source data 2/Figure 1F-ACE2.tif]

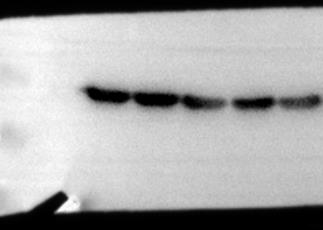

Supplement: Figure 1—source data 2. [file elife-98593-fig1-data2.zip › Figure 1 - Source data 2/Figure 1F-N.tif]

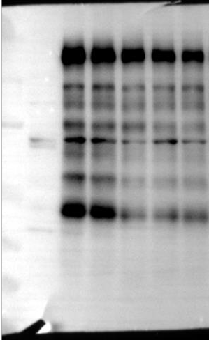

Supplement: Figure 1—source data 2. [file elife-98593-fig1-data2.zip › Figure 1 - Source data 2/Figure 1F-S.tif]

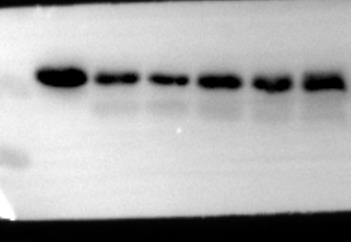

Supplement: Figure 1—source data 2. [file elife-98593-fig1-data2.zip › Figure 1 - Source data 2/Figure 1F-Tubulin.tif]

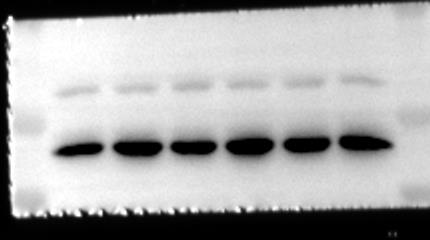

Supplement: Figure 1—source data 2. [file elife-98593-fig1-data2.zip › Figure 1 - Source data 2/Figure 1G-Actin.tif]

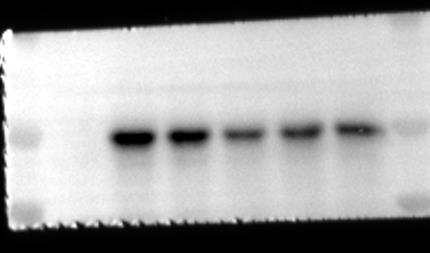

Supplement: Figure 1—source data 2. [file elife-98593-fig1-data2.zip › Figure 1 - Source data 2/Figure 1G-N.tif]

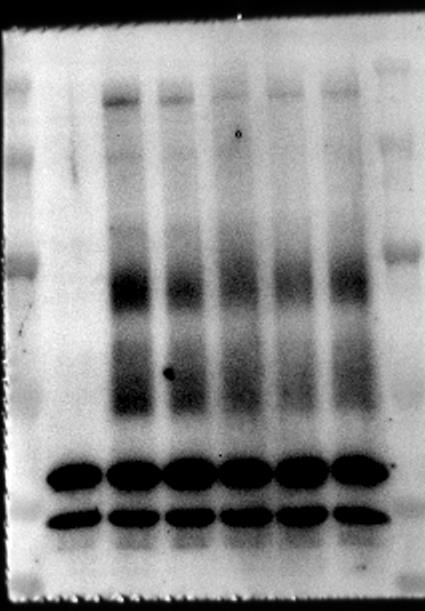

Supplement: Figure 1—source data 2. [file elife-98593-fig1-data2.zip › Figure 1 - Source data 2/Figure 1G-S.tif]

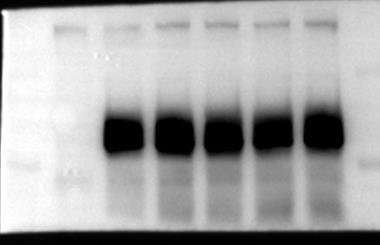

Supplement: Figure 1—figure supplement 1—source data 2. [file elife-98593-fig1-figsupp1-data2.zip › Figure 1–Figure Supplement 1–Source Data 2/Figure 1–Figure Supplement 1C-ACE2.tif]

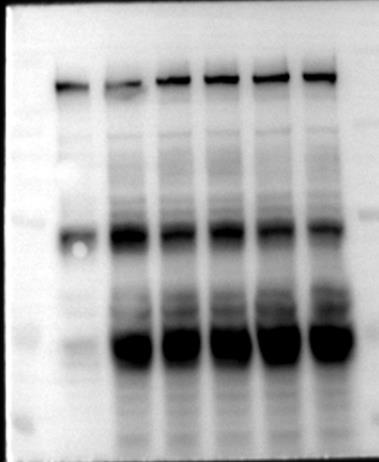

Supplement: Figure 1—figure supplement 1—source data 2. [file elife-98593-fig1-figsupp1-data2.zip › Figure 1–Figure Supplement 1–Source Data 2/Figure 1–Figure Supplement 1C-S.tif]

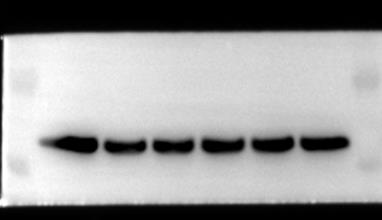

Supplement: Figure 1—figure supplement 1—source data 2. [file elife-98593-fig1-figsupp1-data2.zip › Figure 1–Figure Supplement 1–Source Data 2/Figure 1–Figure Supplement 1C-Tubulin.tif]

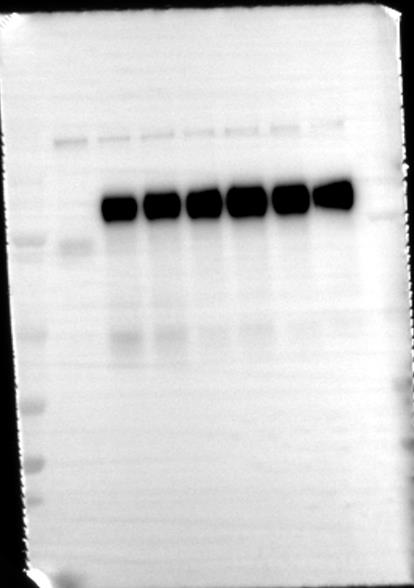

Supplement: Figure 1—figure supplement 1—source data 2. [file elife-98593-fig1-figsupp1-data2.zip › Figure 1–Figure Supplement 1–Source Data 2/Figure 1–Figure Supplement 1G-ACE2.tif]

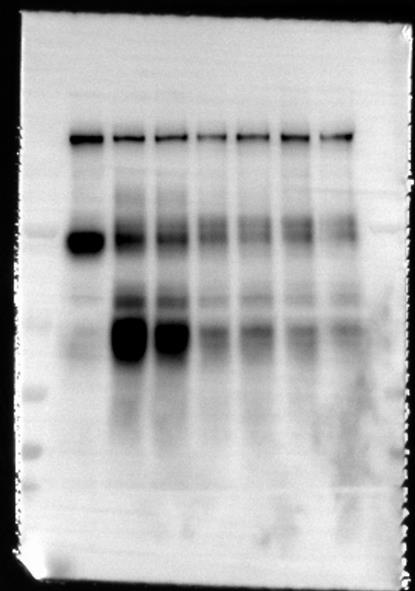

Supplement: Figure 1—figure supplement 1—source data 2. [file elife-98593-fig1-figsupp1-data2.zip › Figure 1–Figure Supplement 1–Source Data 2/Figure 1–Figure Supplement 1G-S.tif]

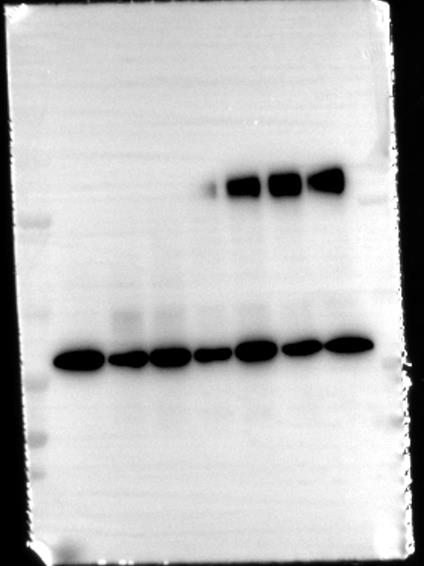

Supplement: Figure 1—figure supplement 1—source data 2. [file elife-98593-fig1-figsupp1-data2.zip › Figure 1–Figure Supplement 1–Source Data 2/Figure 1–Figure Supplement 1G-Tubulin.tif]

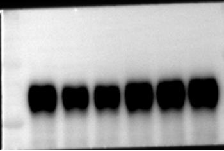

Supplement: Figure 1—figure supplement 2—source data 2. [file elife-98593-fig1-figsupp2-data2.zip › Figure 1–Figure Supplement 2–Source Data 2/Figure 1–Figure Supplement 2A-ACE2.tif]

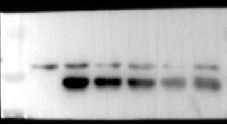

Supplement: Figure 1—figure supplement 2—source data 2. [file elife-98593-fig1-figsupp2-data2.zip › Figure 1–Figure Supplement 2–Source Data 2/Figure 1–Figure Supplement 2A-N.tif]

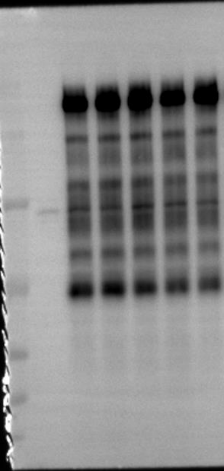

Supplement: Figure 1—figure supplement 2—source data 2. [file elife-98593-fig1-figsupp2-data2.zip › Figure 1–Figure Supplement 2–Source Data 2/Figure 1–Figure Supplement 2A-S.tif]

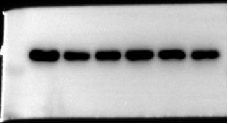

Supplement: Figure 1—figure supplement 2—source data 2. [file elife-98593-fig1-figsupp2-data2.zip › Figure 1–Figure Supplement 2–Source Data 2/Figure 1–Figure Supplement 2A-Tubulin.tif]

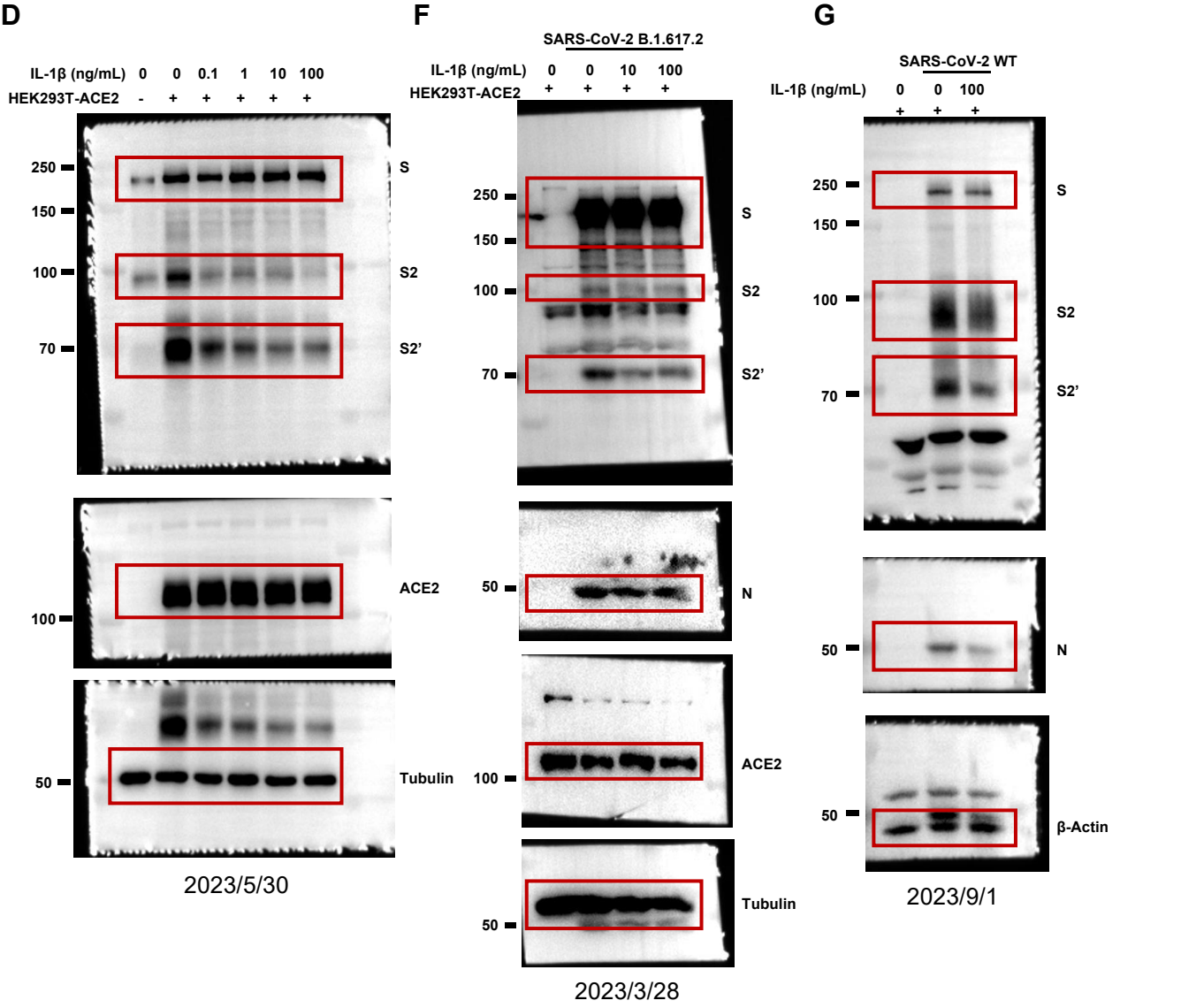

Figure 2-Source Data 1. Original membranes corresponding to Figure 2D, Figure 2F and Figure 2G.

Supplement: Figure 2—source data 1. [file elife-98593-fig2-data1.pdf]

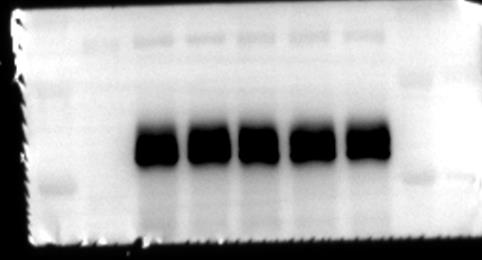

Supplement: Figure 2—source data 2. [file elife-98593-fig2-data2.zip › Figure 2 - Source data 2/Figure 2D-ACE2.tif]

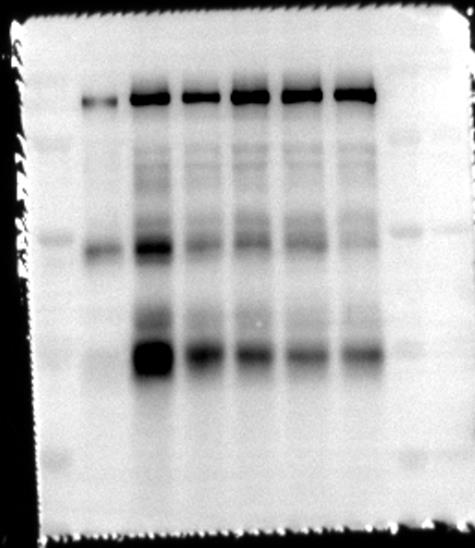

Supplement: Figure 2—source data 2. [file elife-98593-fig2-data2.zip › Figure 2 - Source data 2/Figure 2D-S.tif]

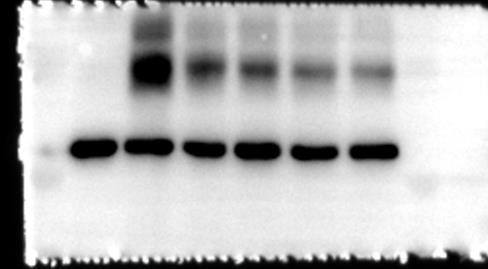

Supplement: Figure 2—source data 2. [file elife-98593-fig2-data2.zip › Figure 2 - Source data 2/Figure 2D-Tubulin.tif]

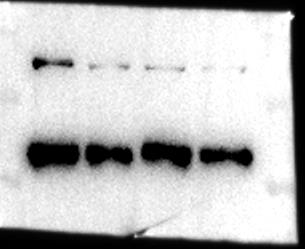

Supplement: Figure 2—source data 2. [file elife-98593-fig2-data2.zip › Figure 2 - Source data 2/Figure 2F-ACE2.tif]

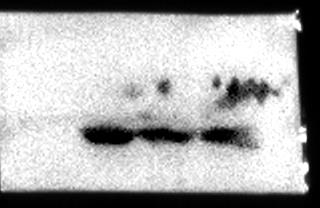

Supplement: Figure 2—source data 2. [file elife-98593-fig2-data2.zip › Figure 2 - Source data 2/Figure 2F-N.tif]

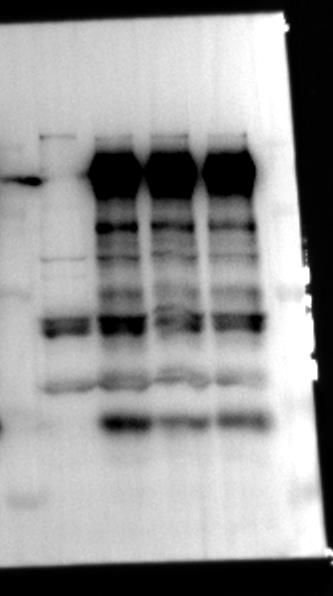

Supplement: Figure 2—source data 2. [file elife-98593-fig2-data2.zip › Figure 2 - Source data 2/Figure 2F-S.tif]

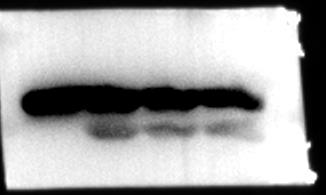

Supplement: Figure 2—source data 2. [file elife-98593-fig2-data2.zip › Figure 2 - Source data 2/Figure 2F-Tubulin.tif]

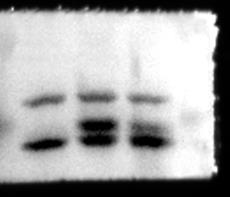

Supplement: Figure 2—source data 2. [file elife-98593-fig2-data2.zip › Figure 2 - Source data 2/Figure 2G-ACTIN.tif]

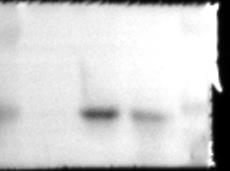

Supplement: Figure 2—source data 2. [file elife-98593-fig2-data2.zip › Figure 2 - Source data 2/Figure 2G-N.tif]

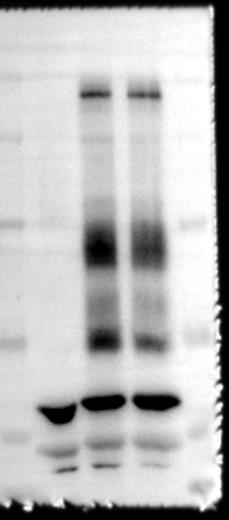

Supplement: Figure 2—source data 2. [file elife-98593-fig2-data2.zip › Figure 2 - Source data 2/Figure 2G-S.tif]

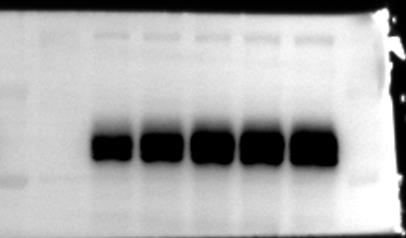

Supplement: Figure 2—figure supplement 2—source data 2. [file elife-98593-fig2-figsupp2-data2.zip › Figure 2–Figure Supplement 2–Source Data 2/Figure 2–Figure Supplement 2A-ACE2.tif]

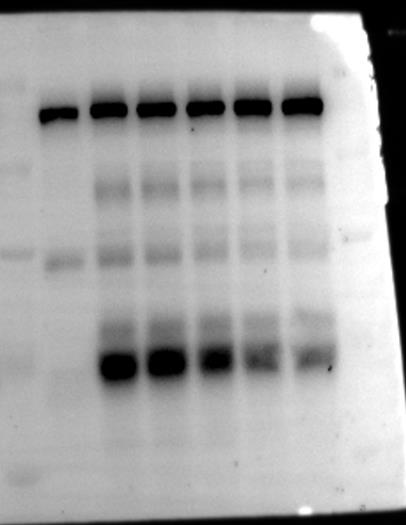

Supplement: Figure 2—figure supplement 2—source data 2. [file elife-98593-fig2-figsupp2-data2.zip › Figure 2–Figure Supplement 2–Source Data 2/Figure 2–Figure Supplement 2A-S.tif]

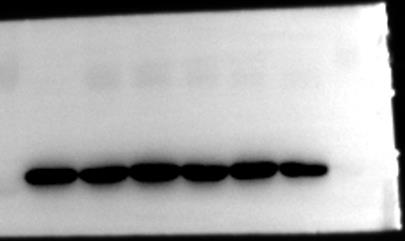

Supplement: Figure 2—figure supplement 2—source data 2. [file elife-98593-fig2-figsupp2-data2.zip › Figure 2–Figure Supplement 2–Source Data 2/Figure 2–Figure Supplement 2A-Tubulin.tif]

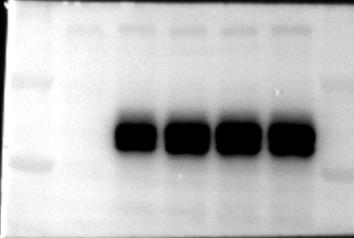

Supplement: Figure 2—figure supplement 2—source data 2. [file elife-98593-fig2-figsupp2-data2.zip › Figure 2–Figure Supplement 2–Source Data 2/Figure 2–Figure Supplement 2F-ACE2.tif]

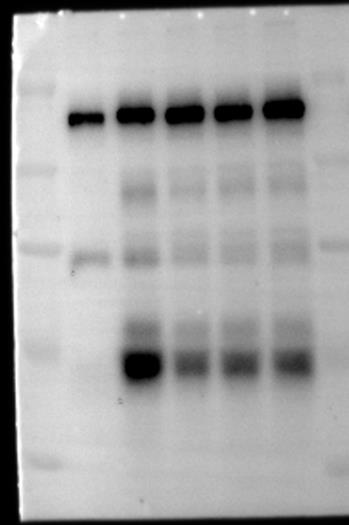

Supplement: Figure 2—figure supplement 2—source data 2. [file elife-98593-fig2-figsupp2-data2.zip › Figure 2–Figure Supplement 2–Source Data 2/Figure 2–Figure Supplement 2F-S.tif]

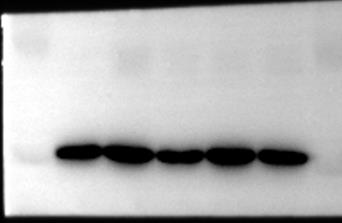

Supplement: Figure 2—figure supplement 2—source data 2. [file elife-98593-fig2-figsupp2-data2.zip › Figure 2–Figure Supplement 2–Source Data 2/Figure 2–Figure Supplement 2F-Tubulin.tif]

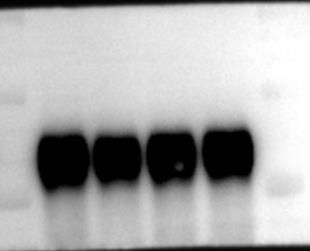

Supplement: Figure 2—figure supplement 3—source data 2. [file elife-98593-fig2-figsupp3-data2.zip › Figure 2–Figure Supplement 3–Source Data 2/Figure 2–Figure Supplement 3A-ACE2.tif]

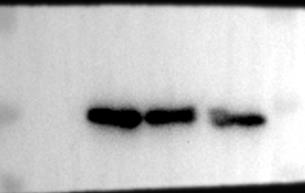

Supplement: Figure 2—figure supplement 3—source data 2. [file elife-98593-fig2-figsupp3-data2.zip › Figure 2–Figure Supplement 3–Source Data 2/Figure 2–Figure Supplement 3A-N.tif]

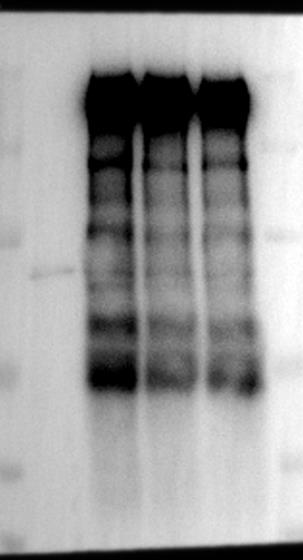

Supplement: Figure 2—figure supplement 3—source data 2. [file elife-98593-fig2-figsupp3-data2.zip › Figure 2–Figure Supplement 3–Source Data 2/Figure 2–Figure Supplement 3A-S.tif]

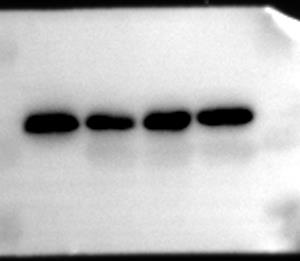

Supplement: Figure 2—figure supplement 3—source data 2. [file elife-98593-fig2-figsupp3-data2.zip › Figure 2–Figure Supplement 3–Source Data 2/Figure 2–Figure Supplement 3A-Tubulin.tif]

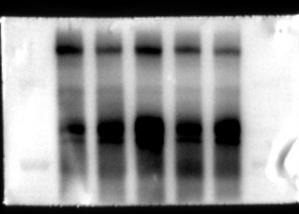

Supplement: Figure 2—figure supplement 4—source data 2. [file elife-98593-fig2-figsupp4-data2.zip › Figure 2–Figure Supplement 4–Source Data 2/Figure 2–Figure Supplement 4C-ACE2.tif]

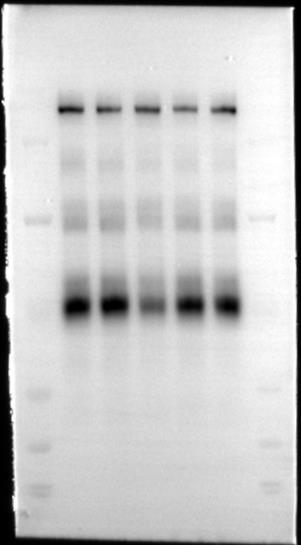

Supplement: Figure 2—figure supplement 4—source data 2. [file elife-98593-fig2-figsupp4-data2.zip › Figure 2–Figure Supplement 4–Source Data 2/Figure 2–Figure Supplement 4C-S.tif]

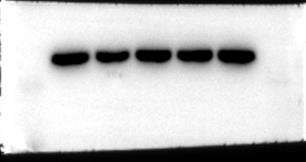

Supplement: Figure 2—figure supplement 4—source data 2. [file elife-98593-fig2-figsupp4-data2.zip › Figure 2–Figure Supplement 4–Source Data 2/Figure 2–Figure Supplement 4C-Tubulin.tif]

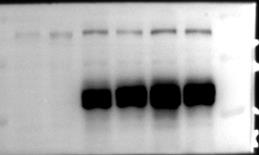

Supplement: Figure 2—figure supplement 5—source data 2. [file elife-98593-fig2-figsupp5-data2.zip › Figure 2–Figure Supplement 5–Source Data 2/Figure 2–Figure Supplement 5B-ACE2.tif]

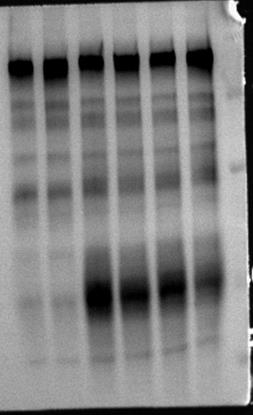

Supplement: Figure 2—figure supplement 5—source data 2. [file elife-98593-fig2-figsupp5-data2.zip › Figure 2–Figure Supplement 5–Source Data 2/Figure 2–Figure Supplement 5B-S.tif]

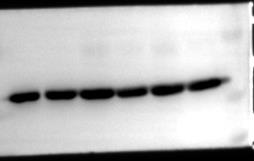

Supplement: Figure 2—figure supplement 5—source data 2. [file elife-98593-fig2-figsupp5-data2.zip › Figure 2–Figure Supplement 5–Source Data 2/Figure 2–Figure Supplement 5B-Tubulin.tif]

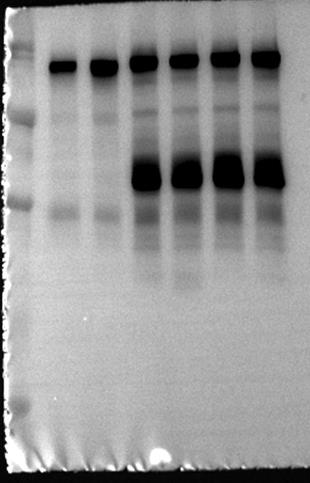

Supplement: Figure 2—figure supplement 5—source data 2. [file elife-98593-fig2-figsupp5-data2.zip › Figure 2–Figure Supplement 5–Source Data 2/Figure 2–Figure Supplement 5C-ACE2.tif]

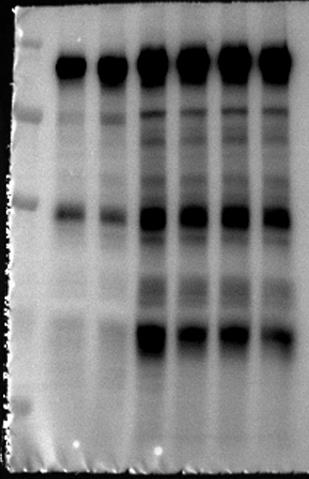

Supplement: Figure 2—figure supplement 5—source data 2. [file elife-98593-fig2-figsupp5-data2.zip › Figure 2–Figure Supplement 5–Source Data 2/Figure 2–Figure Supplement 5C-S.tif]

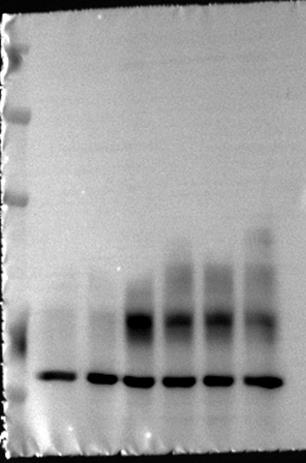

Supplement: Figure 2—figure supplement 5—source data 2. [file elife-98593-fig2-figsupp5-data2.zip › Figure 2–Figure Supplement 5–Source Data 2/Figure 2–Figure Supplement 5C-Tubulin.tif]

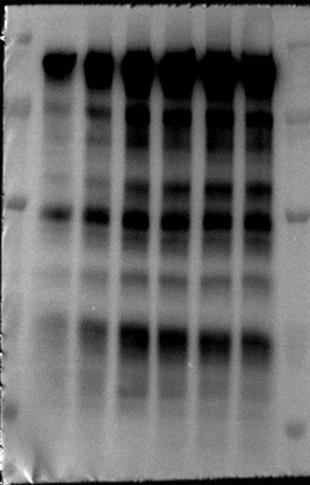

Supplement: Figure 2—figure supplement 5—source data 2. [file elife-98593-fig2-figsupp5-data2.zip › Figure 2–Figure Supplement 5–Source Data 2/Figure 2–Figure Supplement 5D-S.tif]

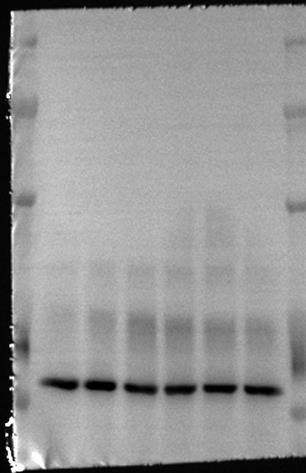

Supplement: Figure 2—figure supplement 5—source data 2. [file elife-98593-fig2-figsupp5-data2.zip › Figure 2–Figure Supplement 5–Source Data 2/Figure 2–Figure Supplement 5D-Tubulin.tif]

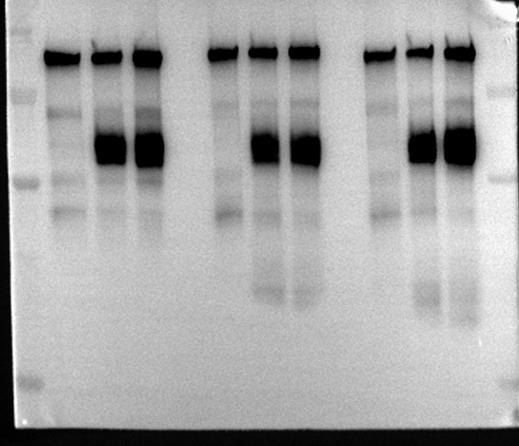

Supplement: Figure 2—figure supplement 6—source data 2. [file elife-98593-fig2-figsupp6-data2.zip › Figure 2–Figure Supplement 6–Source Data 2/Figure 2–Figure Supplement 6D-ACE2.tif]

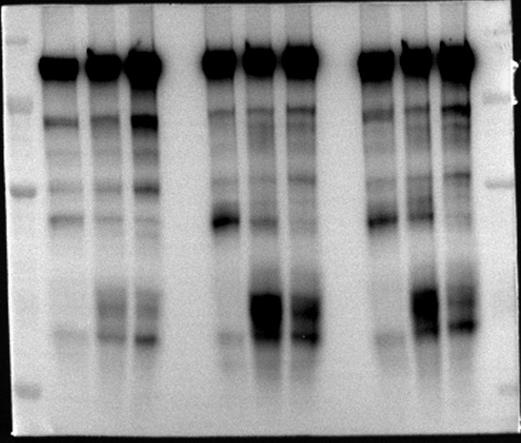

Supplement: Figure 2—figure supplement 6—source data 2. [file elife-98593-fig2-figsupp6-data2.zip › Figure 2–Figure Supplement 6–Source Data 2/Figure 2–Figure Supplement 6D-S.tif]

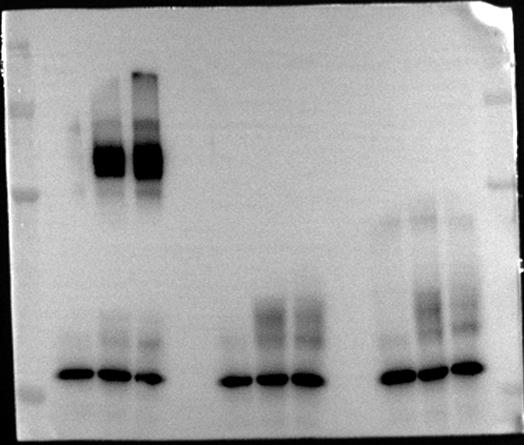

Supplement: Figure 2—figure supplement 6—source data 2. [file elife-98593-fig2-figsupp6-data2.zip › Figure 2–Figure Supplement 6–Source Data 2/Figure 2–Figure Supplement 6D-Tubulin.tif]

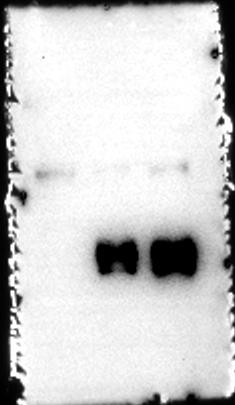

Supplement: Figure 2—figure supplement 6—source data 2. [file elife-98593-fig2-figsupp6-data2.zip › Figure 2–Figure Supplement 6–Source Data 2/Figure 2–Figure Supplement 6E-ACE2.tif]

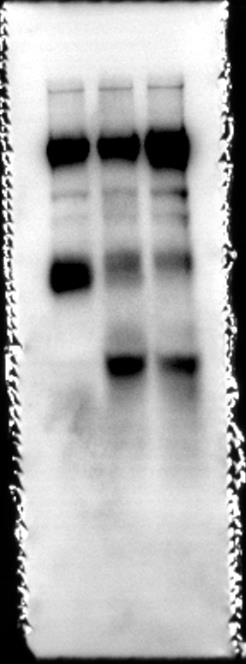

Supplement: Figure 2—figure supplement 6—source data 2. [file elife-98593-fig2-figsupp6-data2.zip › Figure 2–Figure Supplement 6–Source Data 2/Figure 2–Figure Supplement 6E-S.tif]

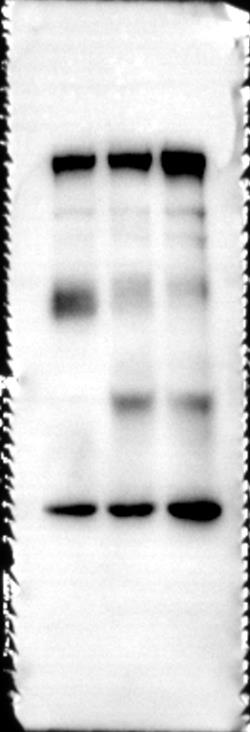

Supplement: Figure 2—figure supplement 6—source data 2. [file elife-98593-fig2-figsupp6-data2.zip › Figure 2–Figure Supplement 6–Source Data 2/Figure 2–Figure Supplement 6E-Tubulin.tif]

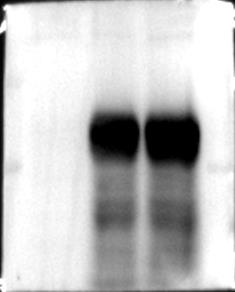

Supplement: Figure 2—figure supplement 7—source data 2. [file elife-98593-fig2-figsupp7-data2.zip › Figure 2–Figure Supplement 7–Source Data 2/Figure 2–Figure Supplement 7D-ACE2.tif]

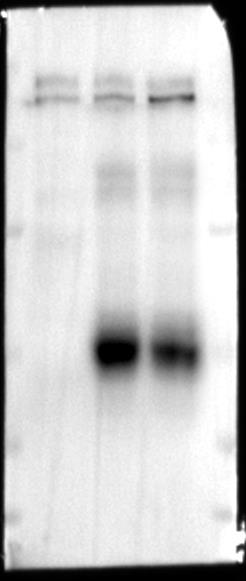

Supplement: Figure 2—figure supplement 7—source data 2. [file elife-98593-fig2-figsupp7-data2.zip › Figure 2–Figure Supplement 7–Source Data 2/Figure 2–Figure Supplement 7D-S.tif]

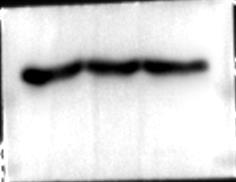

Supplement: Figure 2—figure supplement 7—source data 2. [file elife-98593-fig2-figsupp7-data2.zip › Figure 2–Figure Supplement 7–Source Data 2/Figure 2–Figure Supplement 7D-Tubulin.tif]

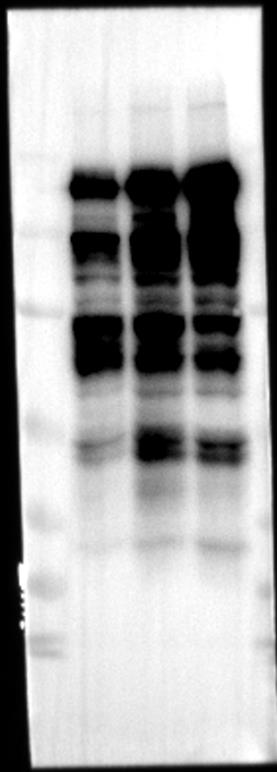

Supplement: Figure 2—figure supplement 7—source data 2. [file elife-98593-fig2-figsupp7-data2.zip › Figure 2–Figure Supplement 7–Source Data 2/Figure 2–Figure Supplement 7E-S.tif]

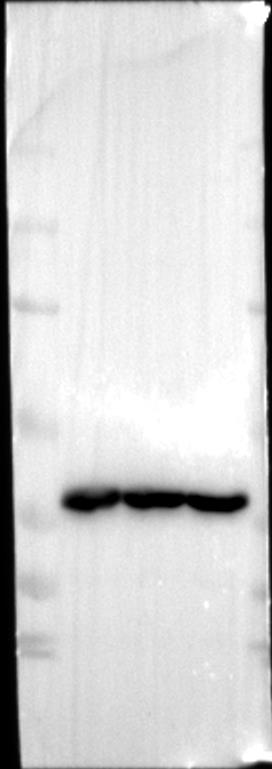

Supplement: Figure 2—figure supplement 7—source data 2. [file elife-98593-fig2-figsupp7-data2.zip › Figure 2–Figure Supplement 7–Source Data 2/Figure 2–Figure Supplement 7E-Tubulin.tif]

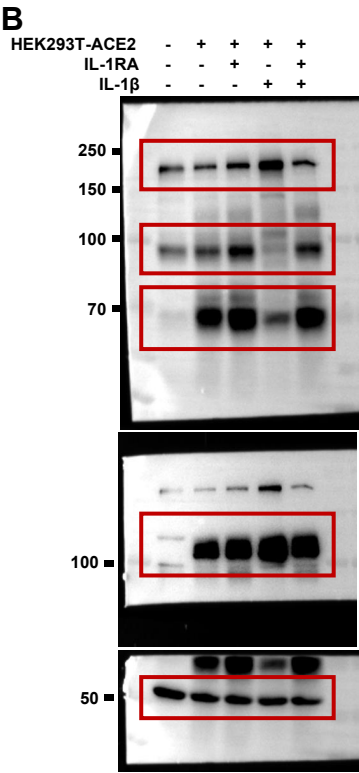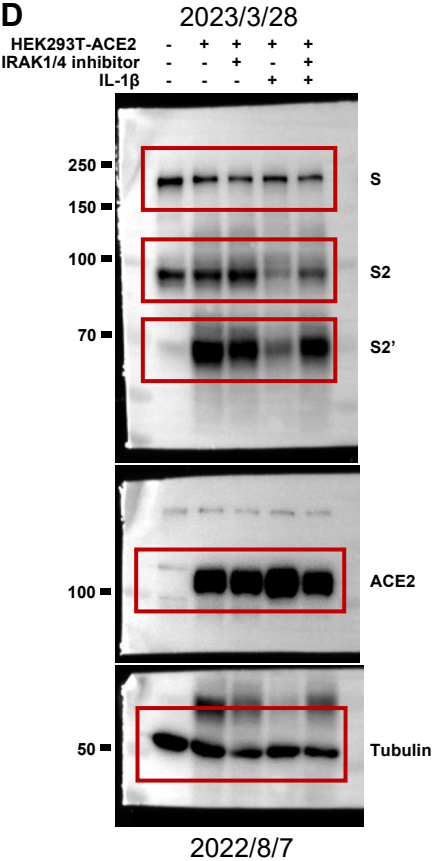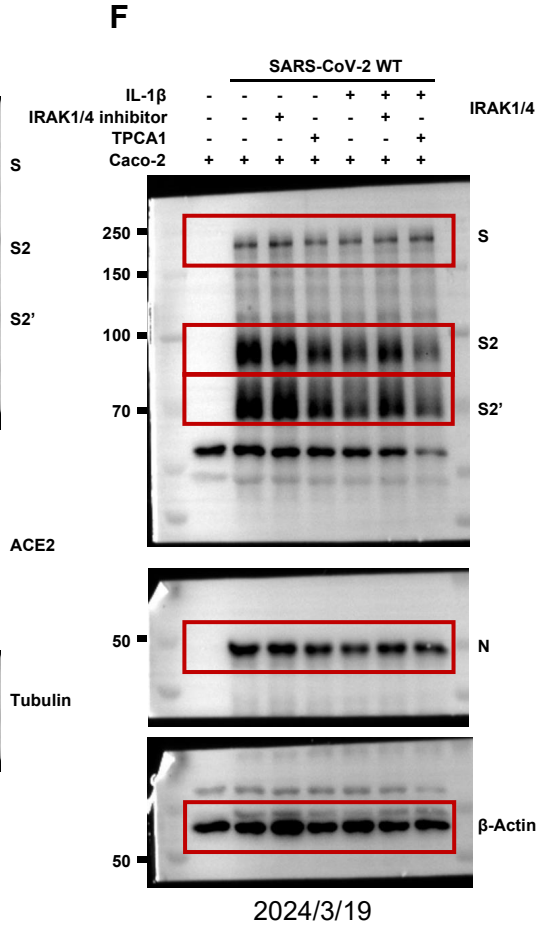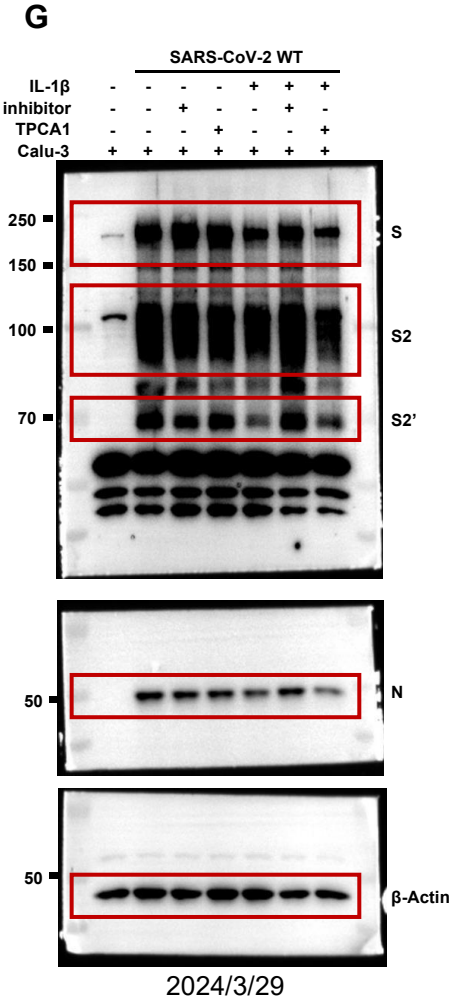

**Figure 3-Source Data 1.** Original membranes corresponding to Figure 3B, Figure 3D, Figure 3F and Figure 3G.

Supplement: Figure 3—source data 1. [file elife-98593-fig3-data1.pdf]

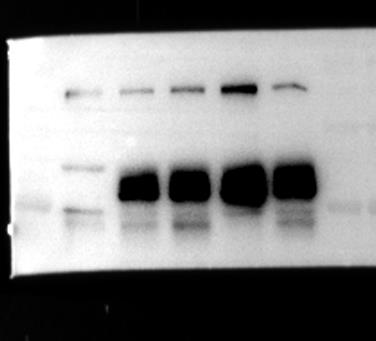

Supplement: Figure 3—source data 2. [file elife-98593-fig3-data2.zip › Figure 3 - Source data 2/Figure 3B-ACE2.tif]

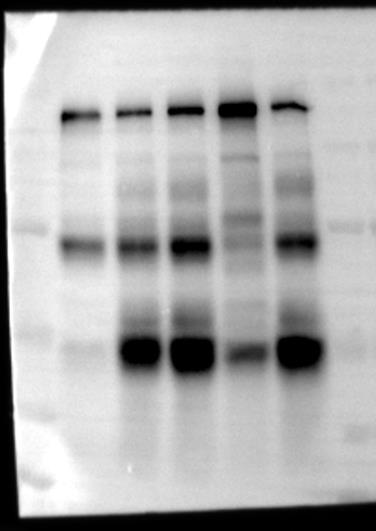

Supplement: Figure 3—source data 2. [file elife-98593-fig3-data2.zip › Figure 3 - Source data 2/Figure 3B-S.tif]

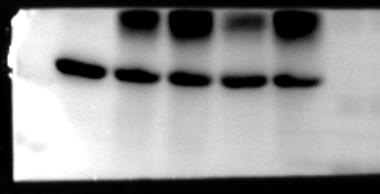

Supplement: Figure 3—source data 2. [file elife-98593-fig3-data2.zip › Figure 3 - Source data 2/Figure 3B-Tubulin.tif]

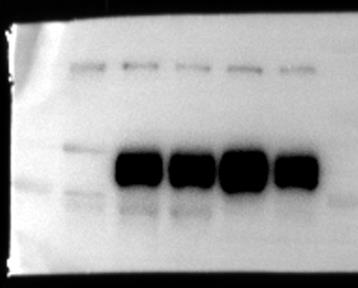

Supplement: Figure 3—source data 2. [file elife-98593-fig3-data2.zip › Figure 3 - Source data 2/Figure 3D-ACE2.tif]

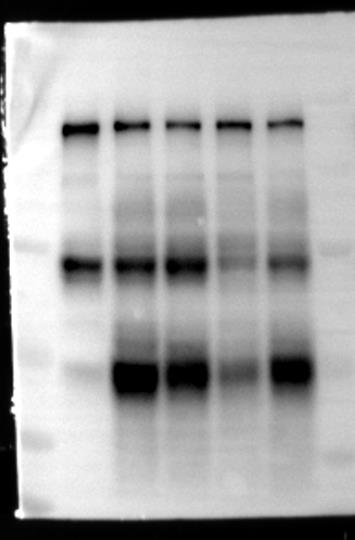

Supplement: Figure 3—source data 2. [file elife-98593-fig3-data2.zip › Figure 3 - Source data 2/Figure 3D-S.tif]

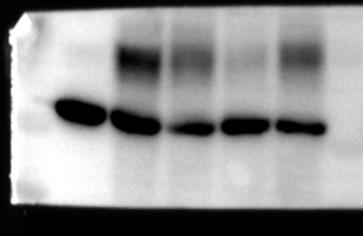

Supplement: Figure 3—source data 2. [file elife-98593-fig3-data2.zip › Figure 3 - Source data 2/Figure 3D-Tubulin.tif]

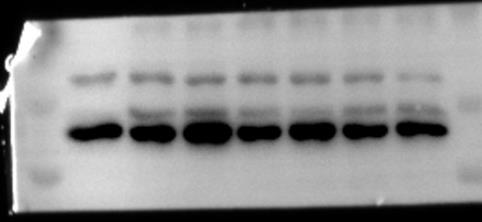

Supplement: Figure 3—source data 2. [file elife-98593-fig3-data2.zip › Figure 3 - Source data 2/Figure 3F-Actin.tif]

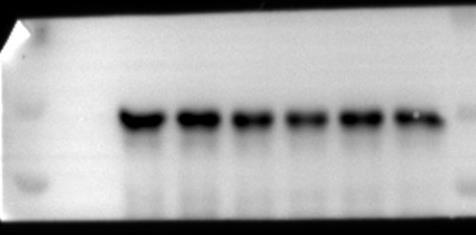

Supplement: Figure 3—source data 2. [file elife-98593-fig3-data2.zip › Figure 3 - Source data 2/Figure 3F-N.tif]

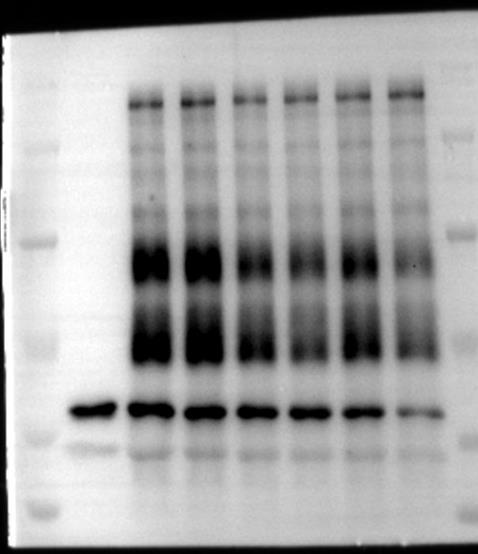

Supplement: Figure 3—source data 2. [file elife-98593-fig3-data2.zip › Figure 3 - Source data 2/Figure 3F-S.tif]

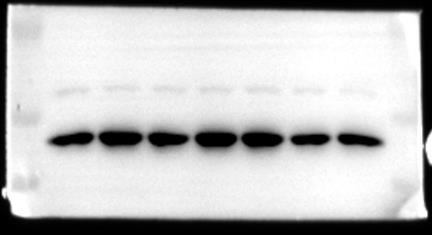

Supplement: Figure 3—source data 2. [file elife-98593-fig3-data2.zip › Figure 3 - Source data 2/Figure 3G-Actin.tif]

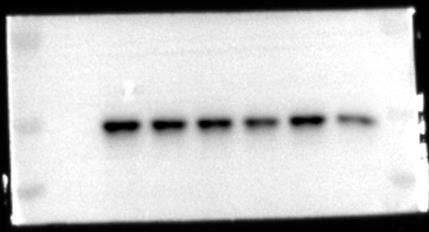

Supplement: Figure 3—source data 2. [file elife-98593-fig3-data2.zip › Figure 3 - Source data 2/Figure 3G-N.tif]

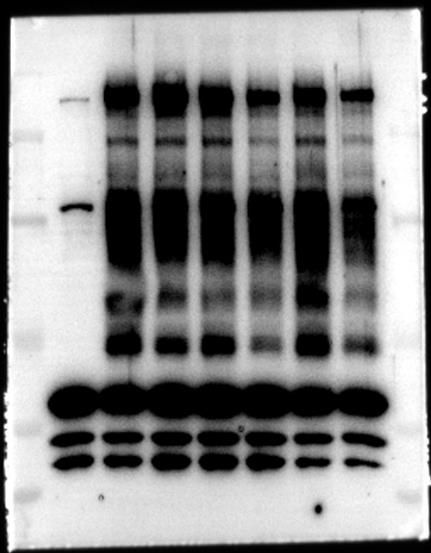

Supplement: Figure 3—source data 2. [file elife-98593-fig3-data2.zip › Figure 3 - Source data 2/Figure 3G-S.tif]

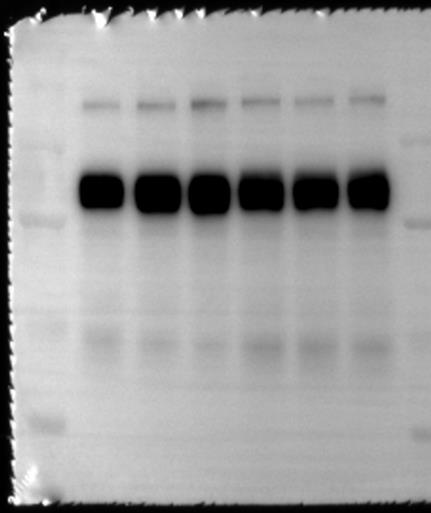

Supplement: Figure 3—figure supplement 1—source data 2. [file elife-98593-fig3-figsupp1-data2.zip › Figure 3–Figure Supplement 1–Source Data 2/Figure 3–Figure Supplement 1A-ACE2.tif]

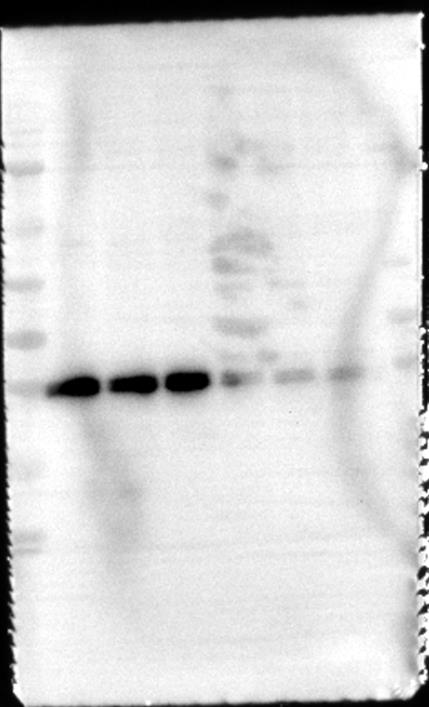

Supplement: Figure 3—figure supplement 1—source data 2. [file elife-98593-fig3-figsupp1-data2.zip › Figure 3–Figure Supplement 1–Source Data 2/Figure 3–Figure Supplement 1A-MyD88.tif]

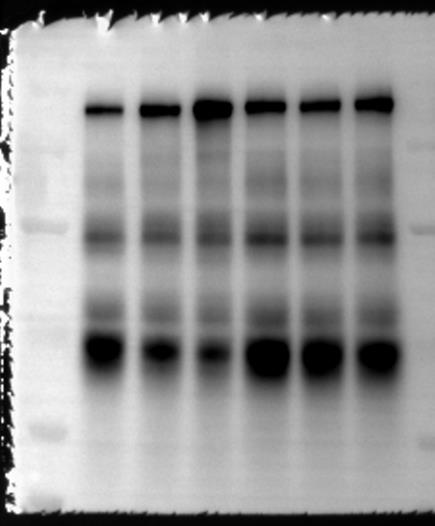

Supplement: Figure 3—figure supplement 1—source data 2. [file elife-98593-fig3-figsupp1-data2.zip › Figure 3–Figure Supplement 1–Source Data 2/Figure 3–Figure Supplement 1A-S.tif]

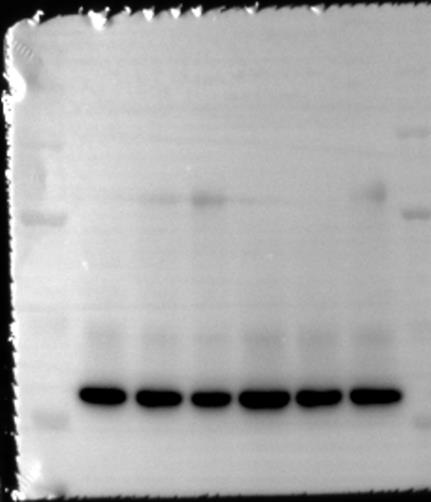

Supplement: Figure 3—figure supplement 1—source data 2. [file elife-98593-fig3-figsupp1-data2.zip › Figure 3–Figure Supplement 1–Source Data 2/Figure 3–Figure Supplement 1A-Tubulin.tif]

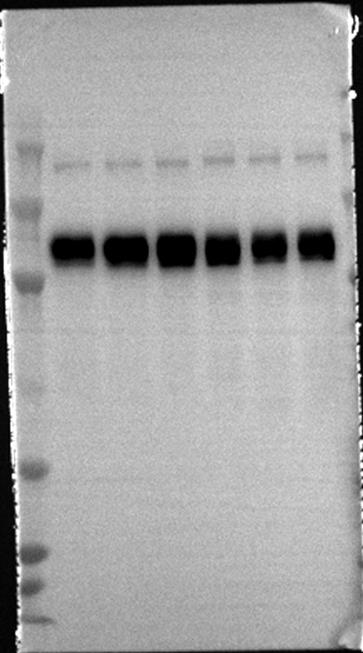

Supplement: Figure 3—figure supplement 1—source data 2. [file elife-98593-fig3-figsupp1-data2.zip › Figure 3–Figure Supplement 1–Source Data 2/Figure 3–Figure Supplement 1B-ACE2.tif]

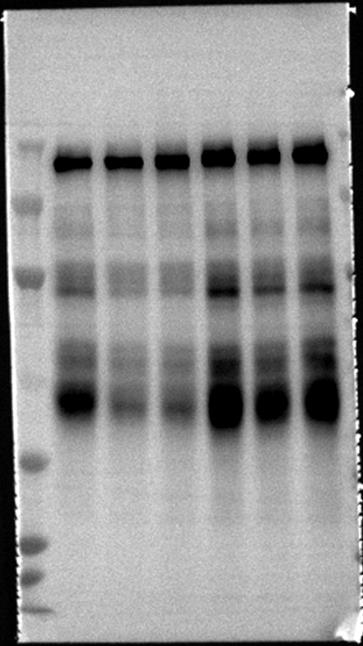

Supplement: Figure 3—figure supplement 1—source data 2. [file elife-98593-fig3-figsupp1-data2.zip › Figure 3–Figure Supplement 1–Source Data 2/Figure 3–Figure Supplement 1B-S.tif]

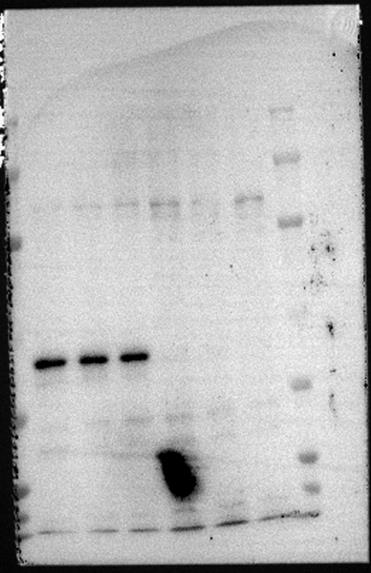

Supplement: Figure 3—figure supplement 1—source data 2. [file elife-98593-fig3-figsupp1-data2.zip › Figure 3–Figure Supplement 1–Source Data 2/Figure 3–Figure Supplement 1B-TRAF6.tif]

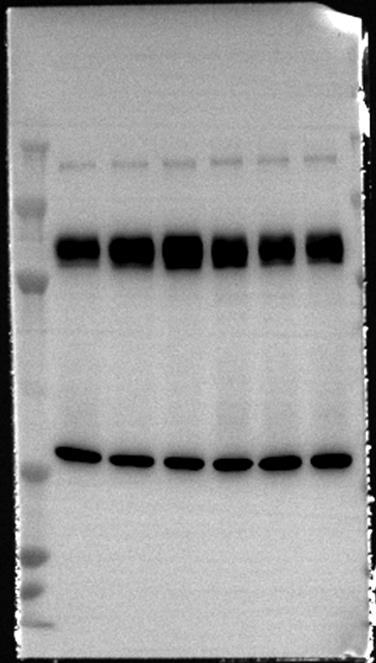

Supplement: Figure 3—figure supplement 1—source data 2. [file elife-98593-fig3-figsupp1-data2.zip › Figure 3–Figure Supplement 1–Source Data 2/Figure 3–Figure Supplement 1B-Tubulin.tif]

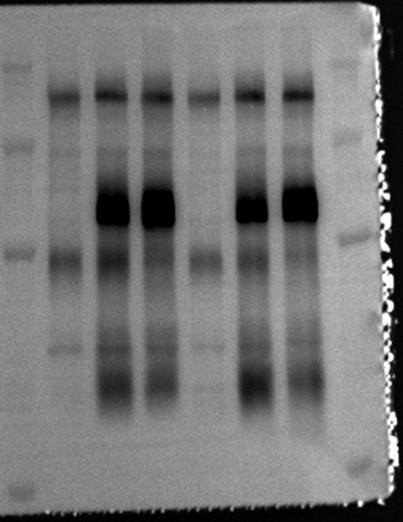

Supplement: Figure 3—figure supplement 2—source data 2. [file elife-98593-fig3-figsupp2-data2.zip › Figure 3–Figure Supplement 2–Source Data 2/Figure 3–Figure Supplement 2A-ACE2.tif]

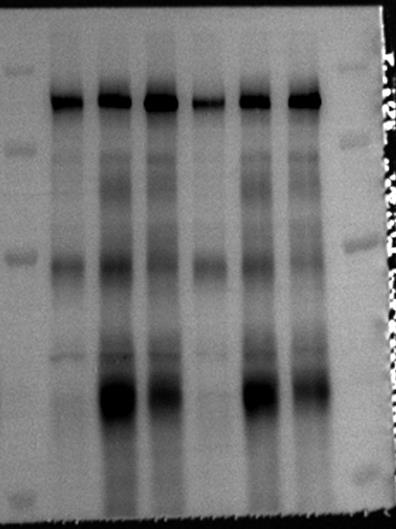

Supplement: Figure 3—figure supplement 2—source data 2. [file elife-98593-fig3-figsupp2-data2.zip › Figure 3–Figure Supplement 2–Source Data 2/Figure 3–Figure Supplement 2A-S.tif]

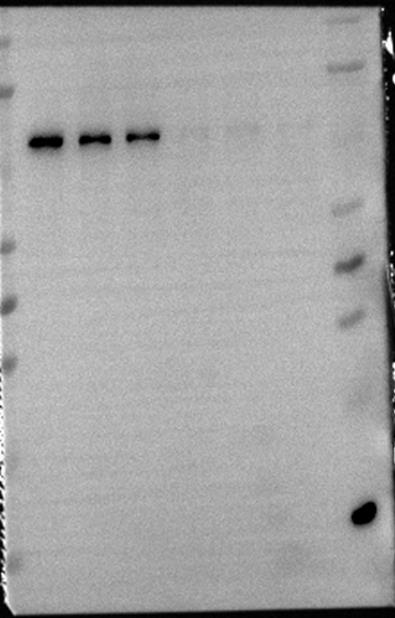

Supplement: Figure 3—figure supplement 2—source data 2. [file elife-98593-fig3-figsupp2-data2.zip › Figure 3–Figure Supplement 2–Source Data 2/Figure 3–Figure Supplement 2A-TAK1.tif]

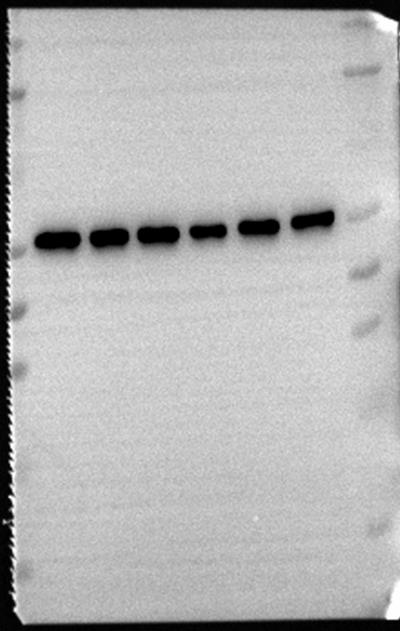

Supplement: Figure 3—figure supplement 2—source data 2. [file elife-98593-fig3-figsupp2-data2.zip › Figure 3–Figure Supplement 2–Source Data 2/Figure 3–Figure Supplement 2A-Tubulin.tif]

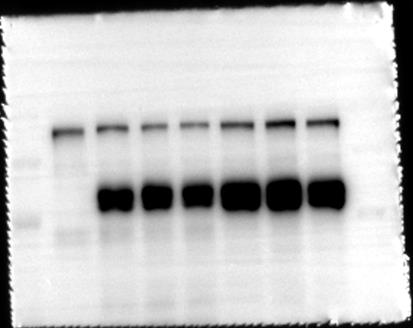

Supplement: Figure 3—figure supplement 2—source data 2. [file elife-98593-fig3-figsupp2-data2.zip › Figure 3–Figure Supplement 2–Source Data 2/Figure 3–Figure Supplement 2B-ACE2.tif]

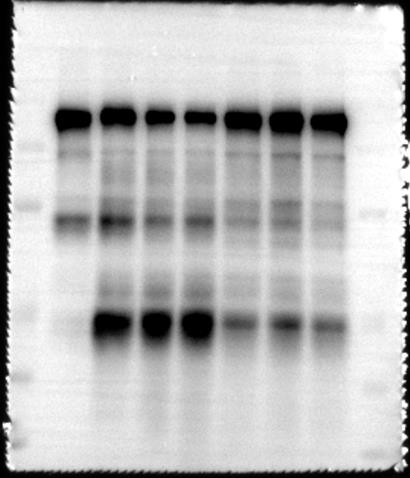

Supplement: Figure 3—figure supplement 2—source data 2. [file elife-98593-fig3-figsupp2-data2.zip › Figure 3–Figure Supplement 2–Source Data 2/Figure 3–Figure Supplement 2B-S.tif]

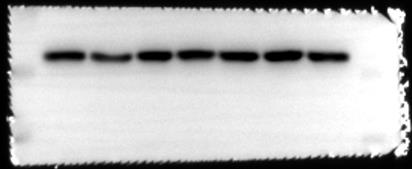

Supplement: Figure 3—figure supplement 2—source data 2. [file elife-98593-fig3-figsupp2-data2.zip › Figure 3–Figure Supplement 2–Source Data 2/Figure 3–Figure Supplement 2B-Tubulin.tif]

A

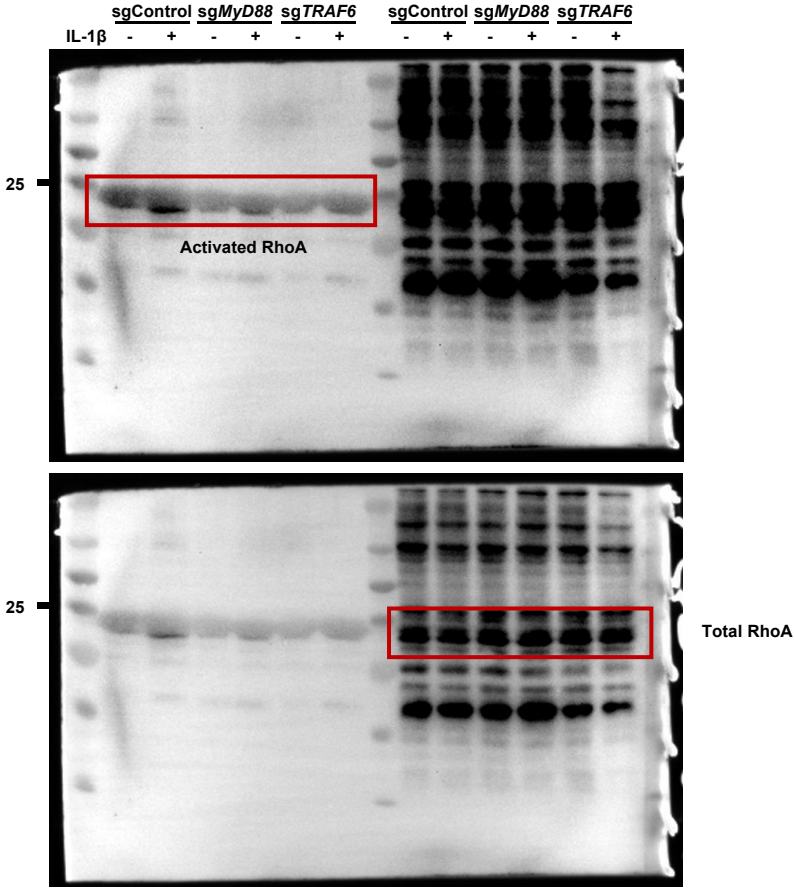

2023/5/20

Figure 4-Source Data 1. Original membranes corresponding to Figure 4A.

Supplement: Figure 4—source data 1. [file elife-98593-fig4-data1.pdf]

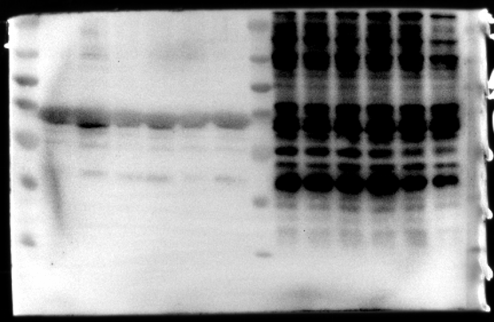

Supplement: Figure 4—source data 2. [file elife-98593-fig4-data2.zip › Figure 4 - Source data 2/Figure 4A-Activated RhoA.tif]

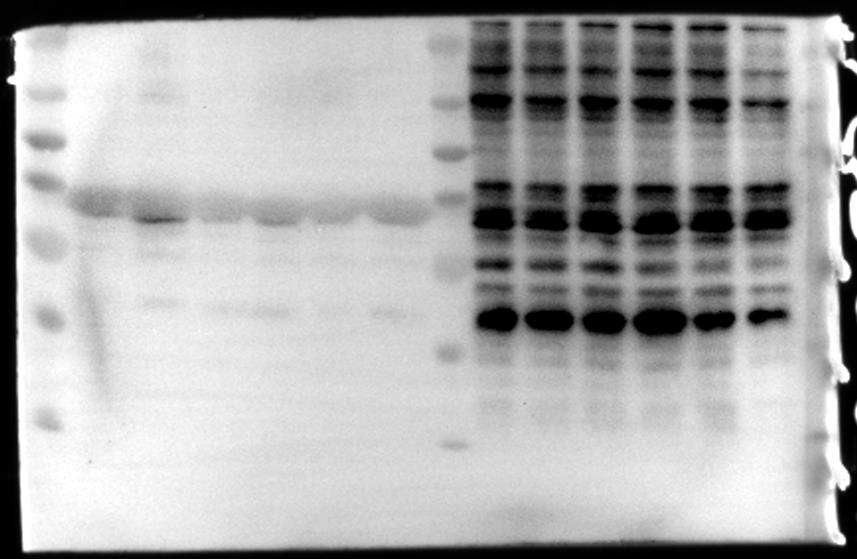

Supplement: Figure 4—source data 2. [file elife-98593-fig4-data2.zip › Figure 4 - Source data 2/Figure 4A-Total RhoA.tif]

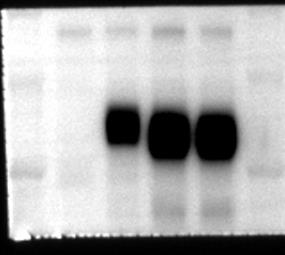

Supplement: Figure 4—figure supplement 2—source data 2. [file elife-98593-fig4-figsupp2-data2.zip › Figure 4–Figure Supplement 2–Source Data 2/Figure 4–Figure Supplement 2A-ACE2.tif]

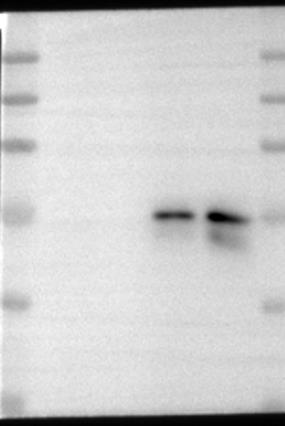

Supplement: Figure 4—figure supplement 2—source data 2. [file elife-98593-fig4-figsupp2-data2.zip › Figure 4–Figure Supplement 2–Source Data 2/Figure 4–Figure Supplement 2A-Myc-RhoA.tif]

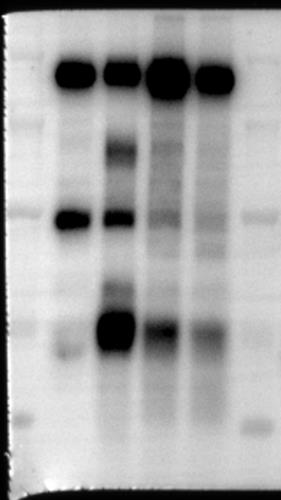

Supplement: Figure 4—figure supplement 2—source data 2. [file elife-98593-fig4-figsupp2-data2.zip › Figure 4–Figure Supplement 2–Source Data 2/Figure 4–Figure Supplement 2A-S.tif]

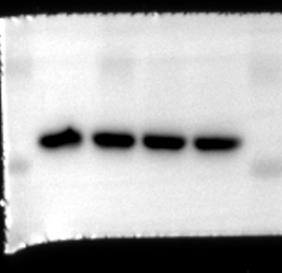

Supplement: Figure 4—figure supplement 2—source data 2. [file elife-98593-fig4-figsupp2-data2.zip › Figure 4–Figure Supplement 2–Source Data 2/Figure 4–Figure Supplement 2A-Tubulin.tif]

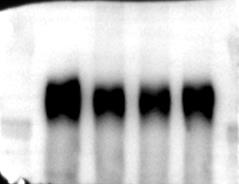

Supplement: Figure 5—source data 2. [file elife-98593-fig5-data2.zip › Figure 5 - Source data 2/Figure 5C-ACE2.tif]
