# Supplementary material for: Interleukin-1 prevents SARS-CoV-2-induced membrane fusion to restrict viral transmission via induction of actin bundles
Source: eLife. 2025 Feb 12;13:RP98593. doi: 10.7554/eLife.98593 (PMC11820142; doi:10.7554/eLife.98593)
Supplement: Figure 1—figure supplement 1—source data 1. [file elife-98593-fig1-figsupp1-data1.pdf]

C

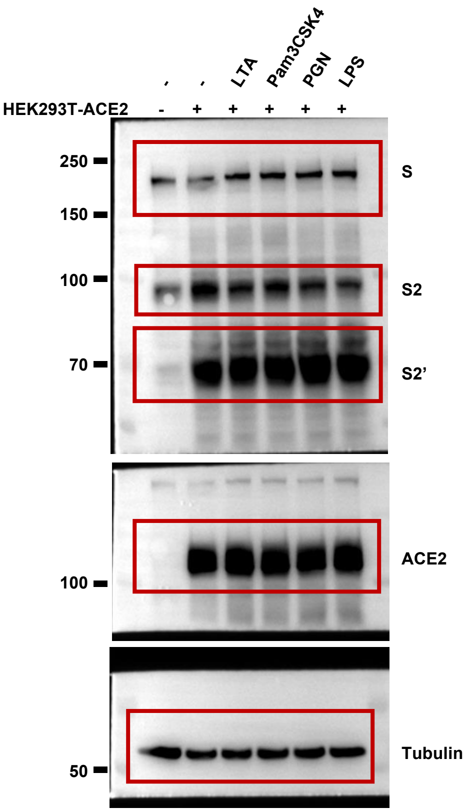

2023/3/26

G

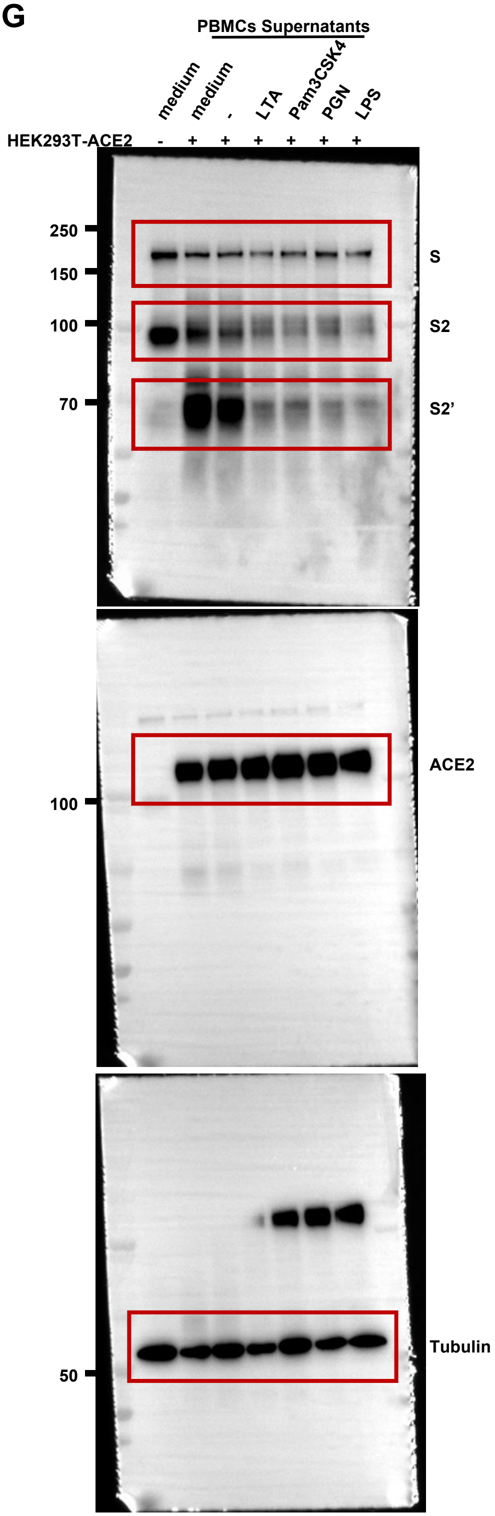

2022/8/5

Figure 1–Figure Supplement 1–Source Data 1. Original membranes corresponding to Figure 1–Figure Supplement 1C and Figure 1–Figure Supplement 1G.
