# Supplementary material for: Interleukin-1 prevents SARS-CoV-2-induced membrane fusion to restrict viral transmission via induction of actin bundles
Source: eLife. 2025 Feb 12;13:RP98593. doi: 10.7554/eLife.98593 (PMC11820142; doi:10.7554/eLife.98593)
Supplement: Figure 2—figure supplement 5—source data 1. [file elife-98593-fig2-figsupp5-data1.pdf]

B

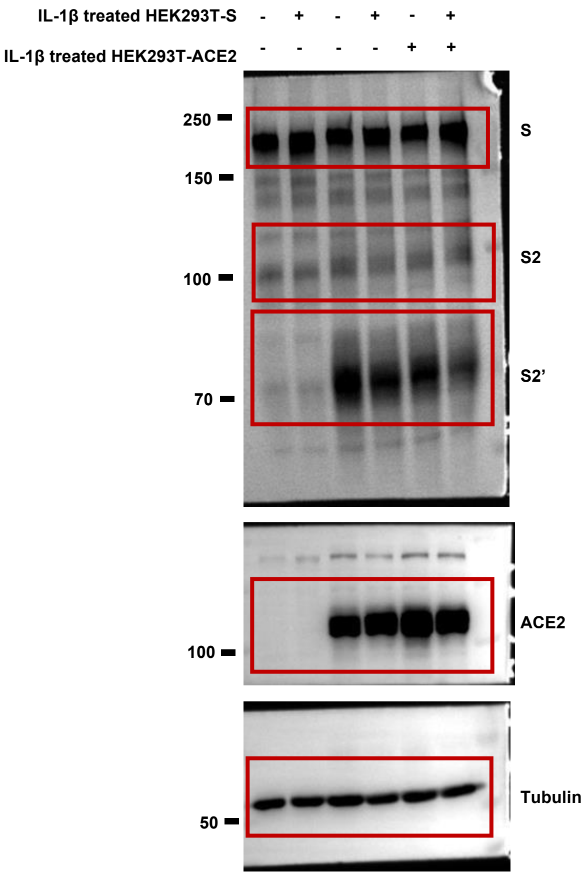

2021/9/27

C

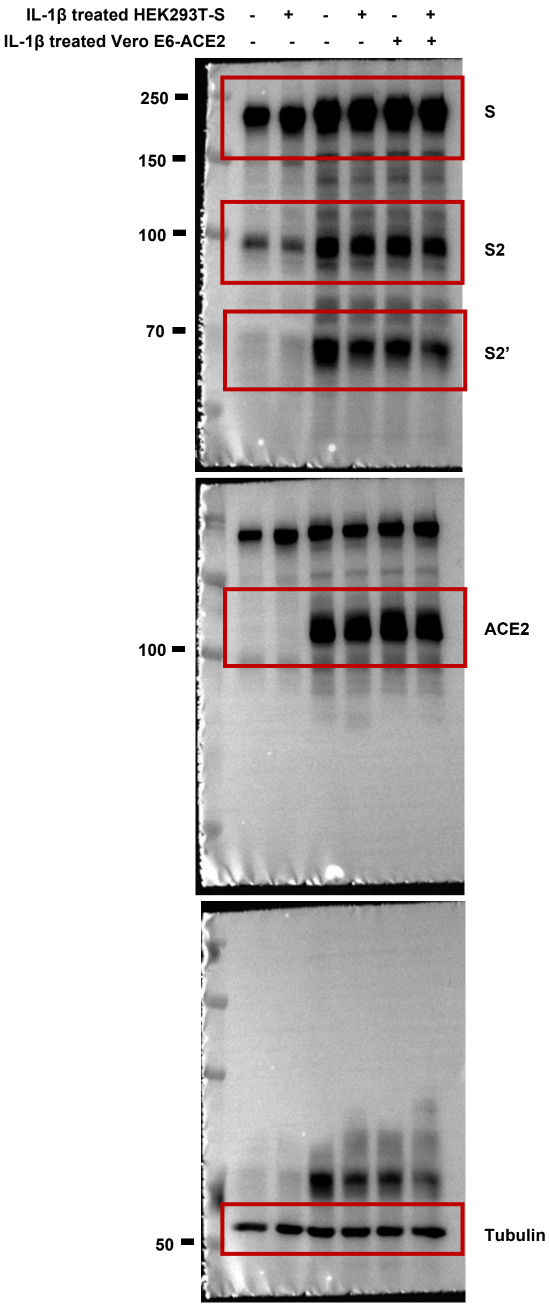

2021/10/12

D

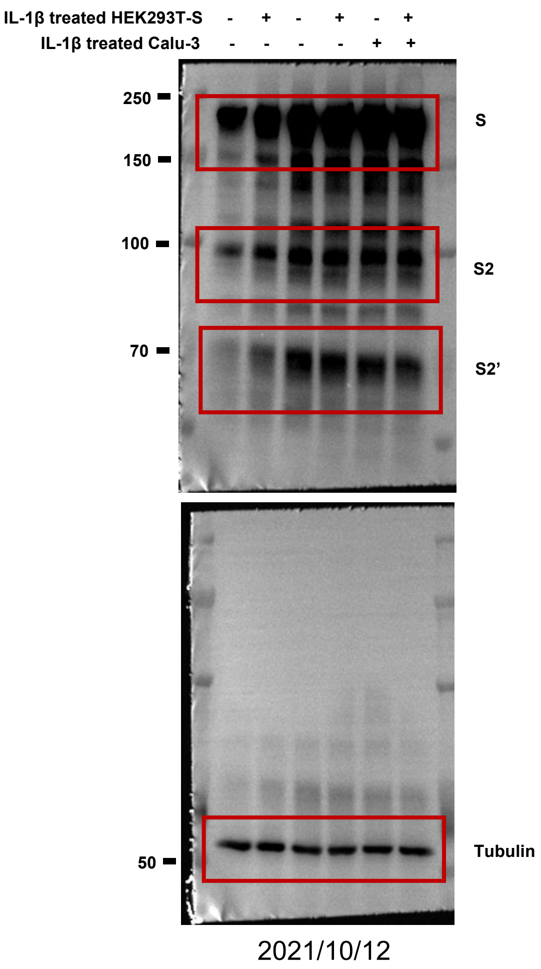

Figure 2–Figure Supplement 5–Source Data 1. Original membranes corresponding to Figure 2–Figure Supplement 5B,Figure 2–Figure Supplement 5C and Figure 2–Figure Supplement 5D.
