# Supplementary material for: Interleukin-1 prevents SARS-CoV-2-induced membrane fusion to restrict viral transmission via induction of actin bundles
Source: eLife. 2025 Feb 12;13:RP98593. doi: 10.7554/eLife.98593 (PMC11820142; doi:10.7554/eLife.98593)
Supplement: Figure 3—figure supplement 1—source data 1. [file elife-98593-fig3-figsupp1-data1.pdf]

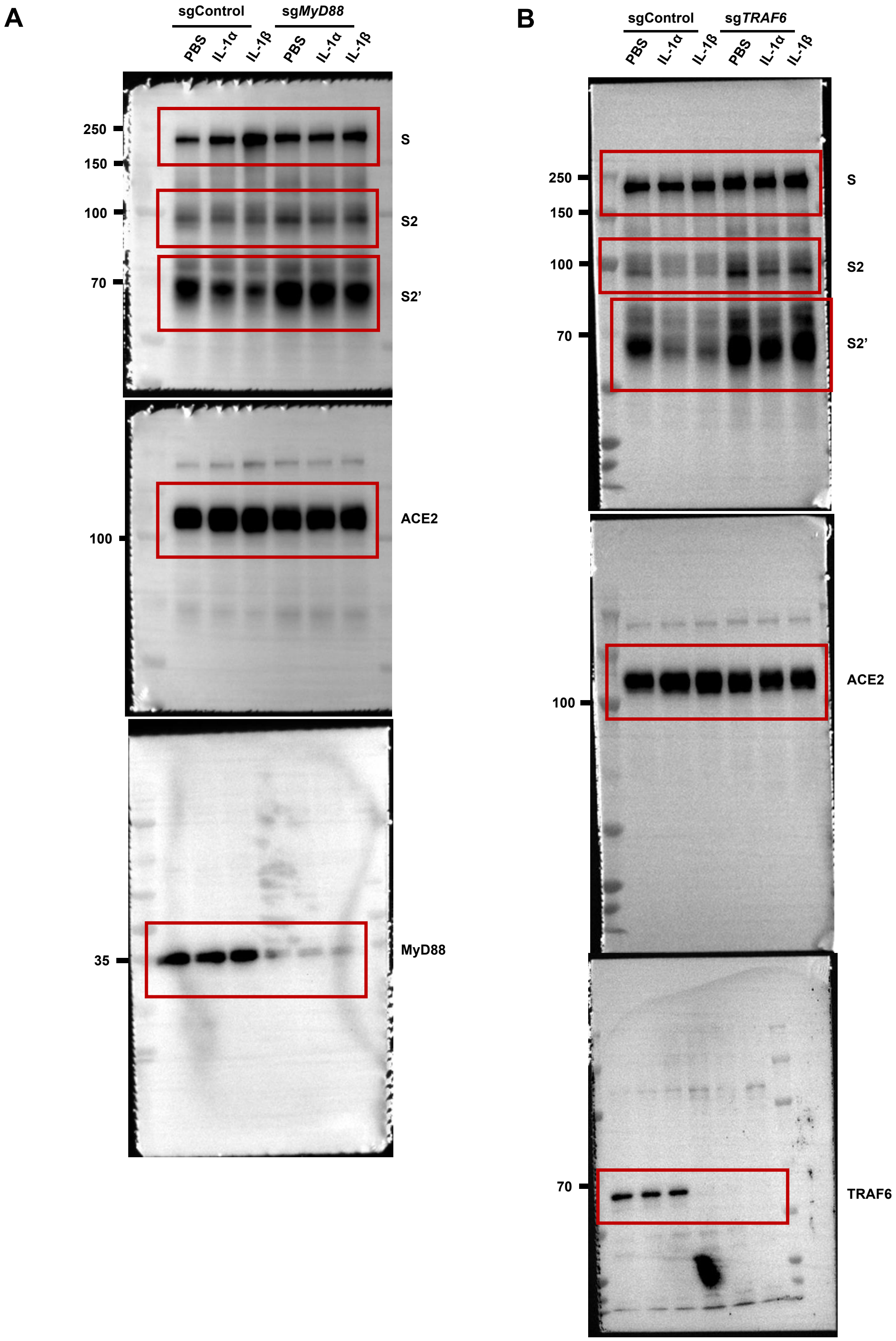

A
sgControl
sgMyD88
PBS
IL-1α
IL-1β
PBS
IL-1α
IL-1β
250
150
100
70
S
S2
S2'
100
ACE2
35
MyD88
B
sgControl
sgTRAF6
PBS
IL-1α
IL-1β
PBS
IL-1α
IL-1β
250
150
100
70
S
S2
S2'
100
ACE2
70
TRAF6

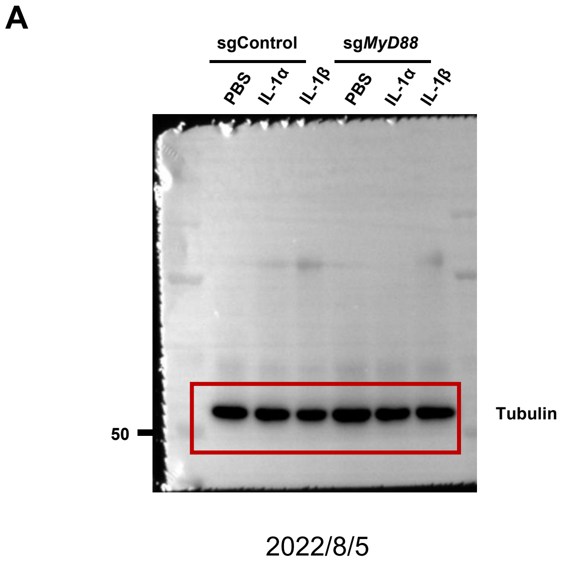

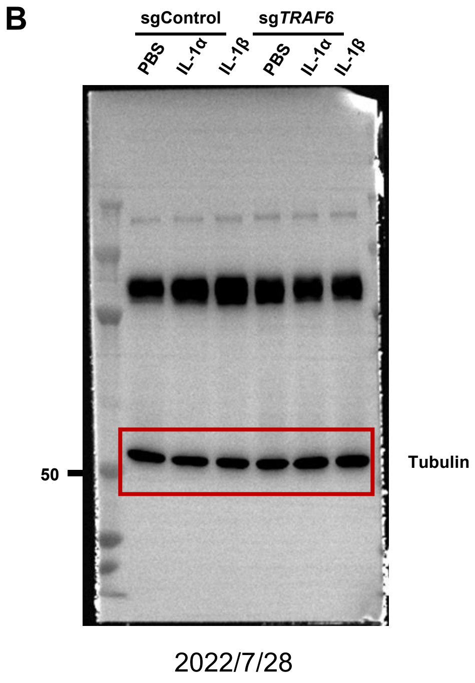

Figure 3–Figure Supplement 1–Source Data 1. Original membranes corresponding to Figure 3–Figure Supplement 1A and Figure 3–Figure Supplement 1B.
