# Supplementary material for: Interleukin-1 prevents SARS-CoV-2-induced membrane fusion to restrict viral transmission via induction of actin bundles
Source: eLife. 2025 Feb 12;13:RP98593. doi: 10.7554/eLife.98593 (PMC11820142; doi:10.7554/eLife.98593)
Supplement: Figure 3—figure supplement 2—source data 1. [file elife-98593-fig3-figsupp2-data1.pdf]

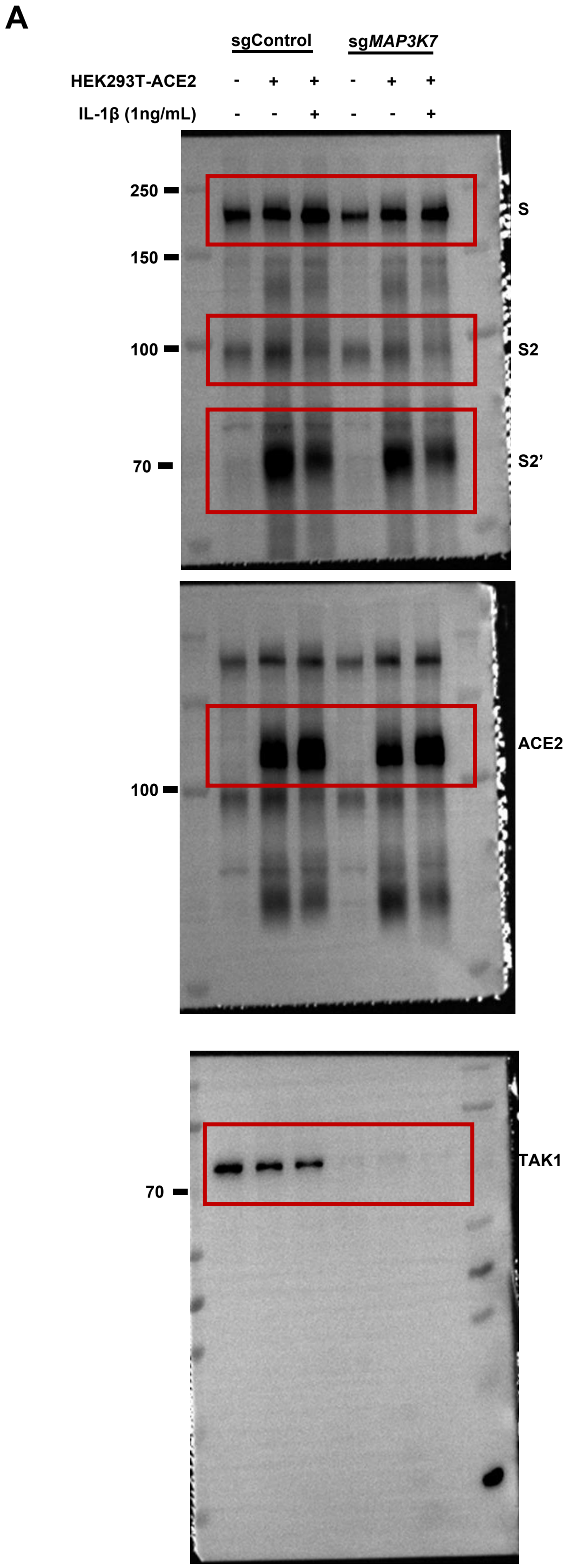
A
sgControl
sgMAP3K7
HEK293T-ACE2 - + + - + +
IL-1β (1ng/mL) - - + - - +
250
150
100
70
S
S2
S2'
100
ACE2
70
TAK1

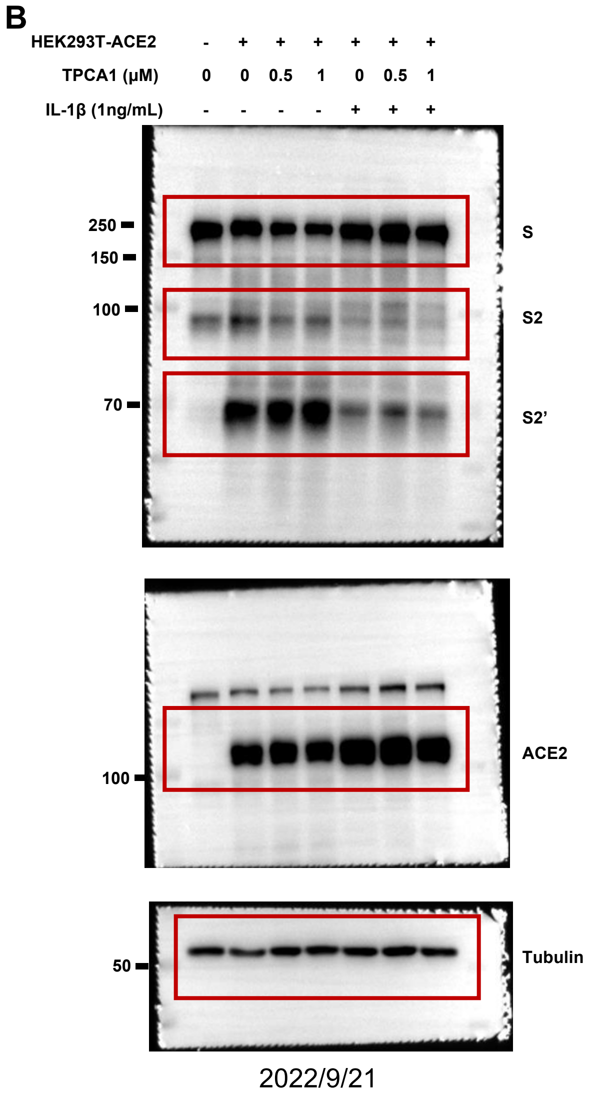
B
HEK293T-ACE2 - + + + + + +
TPCA1 (μM) 0 0 0.5 1 0 0.5 1
IL-1β (1ng/mL) - - - - + + +
250
150
100
70
S
S2
S2'
100
ACE2
50
Tubulin
2022/9/21

A

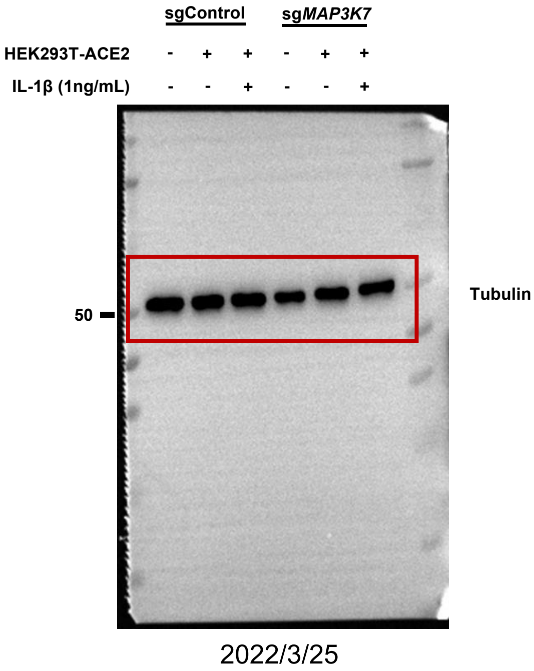

Figure 3–Figure Supplement 2–Source Data 1. Original membranes corresponding to Figure 3–Figure Supplement 2A and Figure 3–Figure Supplement 2B.
