# Supplementary material for: Interleukin-1 prevents SARS-CoV-2-induced membrane fusion to restrict viral transmission via induction of actin bundles
Source: eLife. 2025 Feb 12;13:RP98593. doi: 10.7554/eLife.98593 (PMC11820142; doi:10.7554/eLife.98593)
Supplement: Figure 5—source data 1. [file elife-98593-fig5-data1.pdf]

C

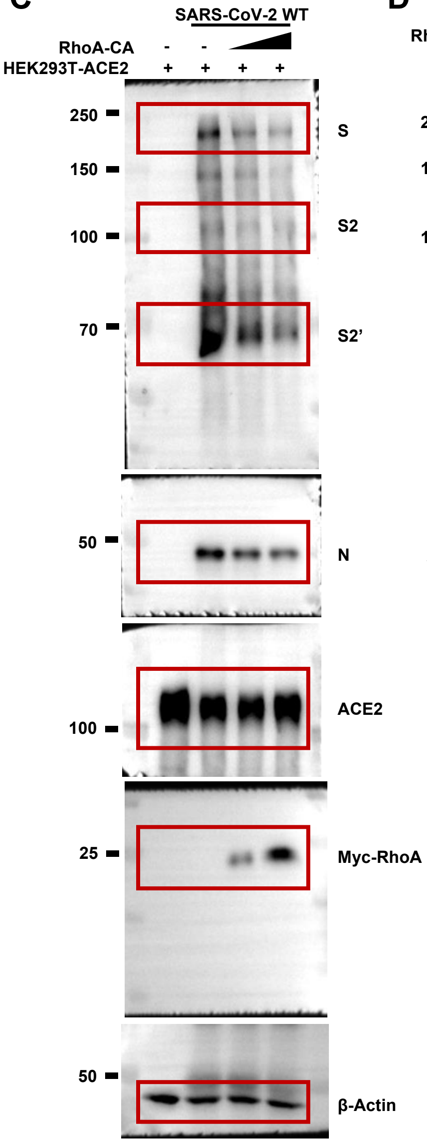

2023/9/1

D

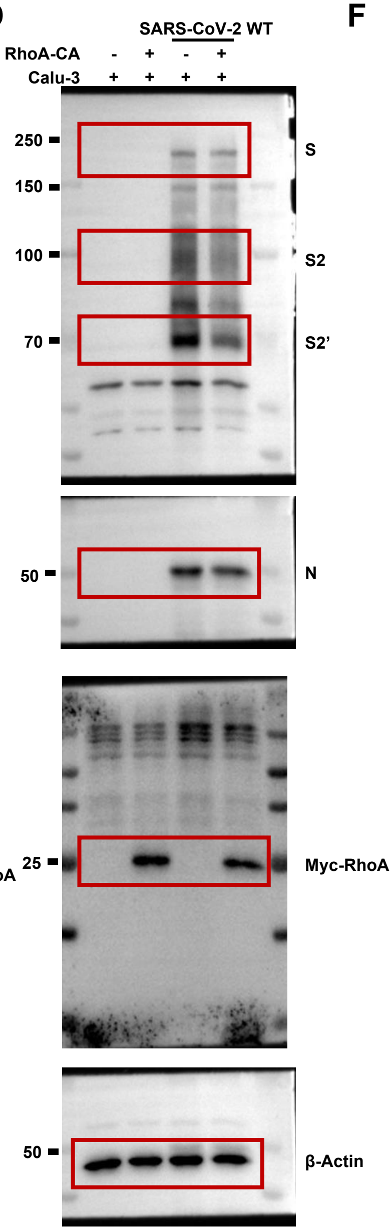

2024/3/19

F

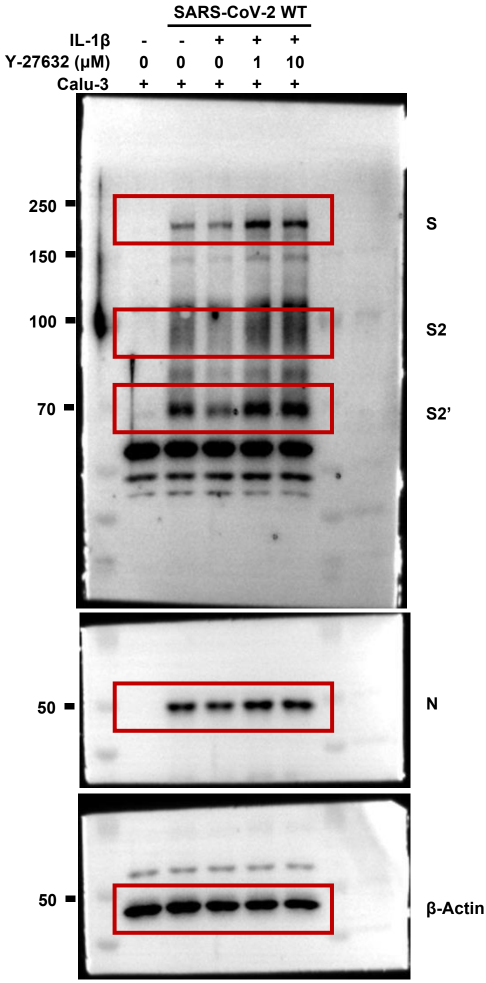

2024/3/26

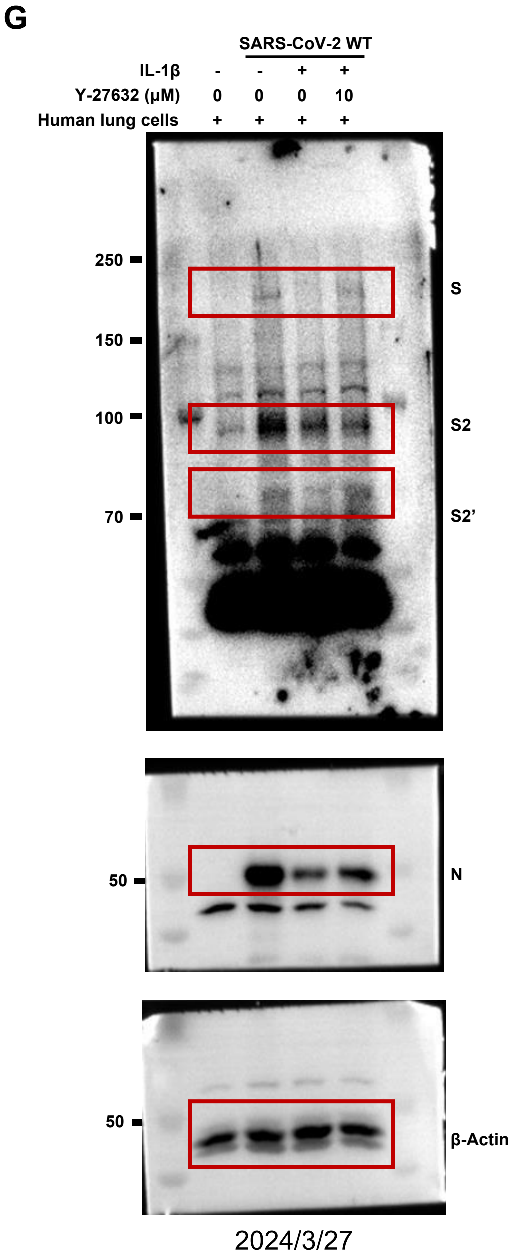

Figure 5-Source Data 1. Original membranes corresponding to Figure 5C, Figure 5D, Figure 5F and Figure 5G.
