# Supplementary figures and images for: Interleukin-1 prevents SARS-CoV-2-induced membrane fusion to restrict viral transmission via induction of actin bundles (part 2 of 2)
Source: eLife. 2025 Feb 12;13:RP98593. doi: 10.7554/eLife.98593 (PMC11820142; doi:10.7554/eLife.98593)

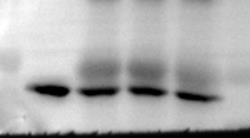

Supplement: Figure 5—source data 2. [file elife-98593-fig5-data2.zip › Figure 5 - Source data 2/Figure 5C-Actin.tif]

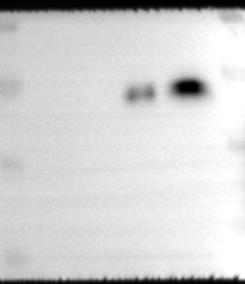

Supplement: Figure 5—source data 2. [file elife-98593-fig5-data2.zip › Figure 5 - Source data 2/Figure 5C-Myc-RhoA.tif]

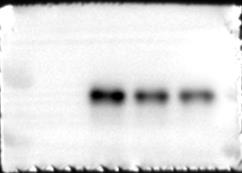

Supplement: Figure 5—source data 2. [file elife-98593-fig5-data2.zip › Figure 5 - Source data 2/Figure 5C-N.tif]

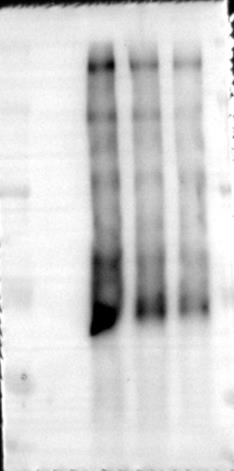

Supplement: Figure 5—source data 2. [file elife-98593-fig5-data2.zip › Figure 5 - Source data 2/Figure 5C-S.tif]

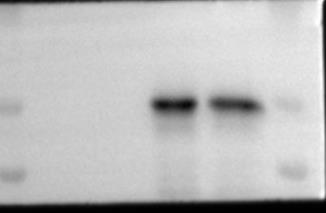

Supplement: Figure 5—source data 2. [file elife-98593-fig5-data2.zip › Figure 5 - Source data 2/Figure 5D-ACE2.tif]

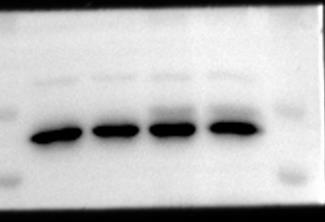

Supplement: Figure 5—source data 2. [file elife-98593-fig5-data2.zip › Figure 5 - Source data 2/Figure 5D-Actin.tif]

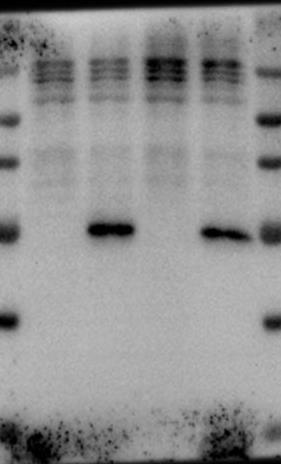

Supplement: Figure 5—source data 2. [file elife-98593-fig5-data2.zip › Figure 5 - Source data 2/Figure 5D-Myc-RhoA.tif]

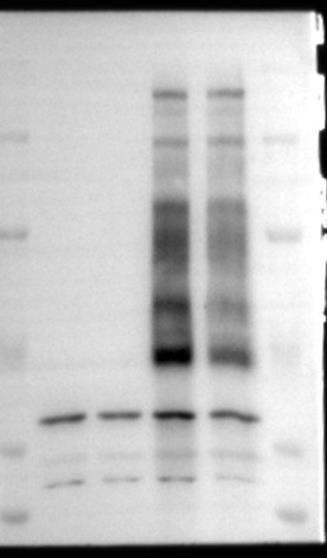

Supplement: Figure 5—source data 2. [file elife-98593-fig5-data2.zip › Figure 5 - Source data 2/Figure 5D-S.tif]

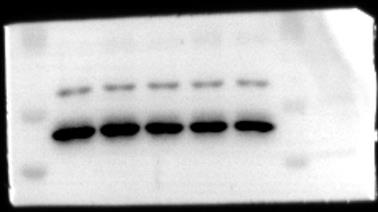

Supplement: Figure 5—source data 2. [file elife-98593-fig5-data2.zip › Figure 5 - Source data 2/Figure 5F-Actin.tif]

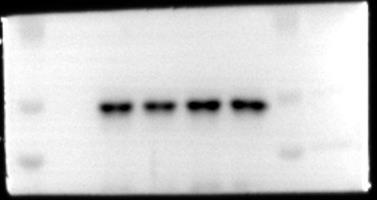

Supplement: Figure 5—source data 2. [file elife-98593-fig5-data2.zip › Figure 5 - Source data 2/Figure 5F-N.tif]

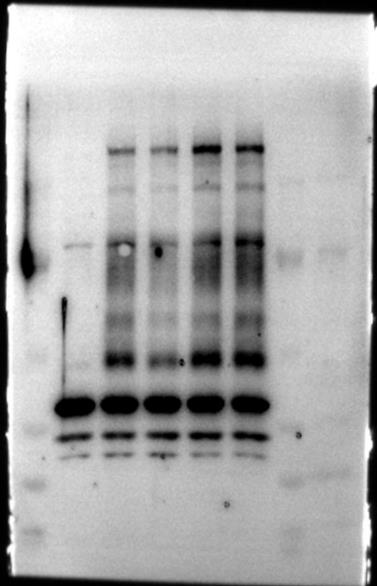

Supplement: Figure 5—source data 2. [file elife-98593-fig5-data2.zip › Figure 5 - Source data 2/Figure 5F-S.tif]

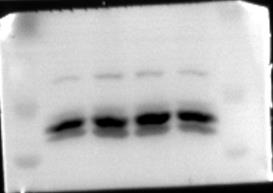

Supplement: Figure 5—source data 2. [file elife-98593-fig5-data2.zip › Figure 5 - Source data 2/Figure 5G-Actin.tif]

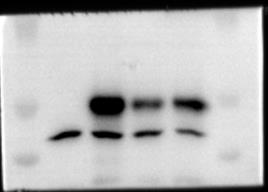

Supplement: Figure 5—source data 2. [file elife-98593-fig5-data2.zip › Figure 5 - Source data 2/Figure 5G-N.tif]

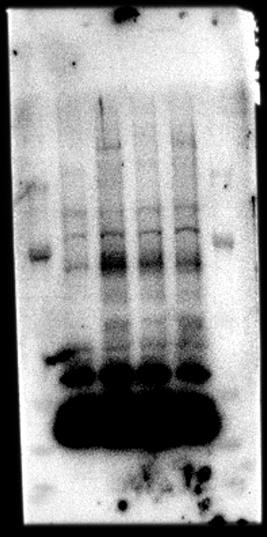

Supplement: Figure 5—source data 2. [file elife-98593-fig5-data2.zip › Figure 5 - Source data 2/Figure 5G-S.tif]

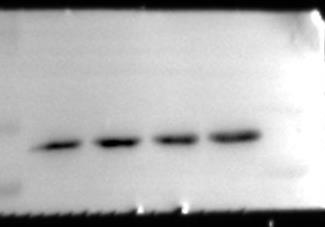

Supplement: Figure 5—figure supplement 2—source data 2. [file elife-98593-fig5-figsupp2-data2.zip › Figure 5–Figure Supplement 2–Source Data 2/Figure 5–Figure Supplement 2A-Actin.tif]

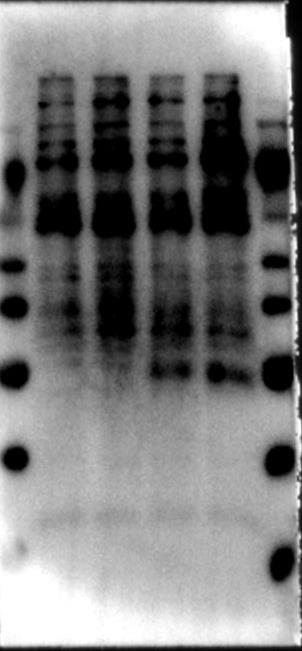

Supplement: Figure 5—figure supplement 2—source data 2. [file elife-98593-fig5-figsupp2-data2.zip › Figure 5–Figure Supplement 2–Source Data 2/Figure 5–Figure Supplement 2A-Myc-RhoA.tif]

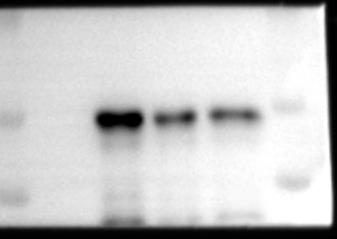

Supplement: Figure 5—figure supplement 2—source data 2. [file elife-98593-fig5-figsupp2-data2.zip › Figure 5–Figure Supplement 2–Source Data 2/Figure 5–Figure Supplement 2A-N.tif]

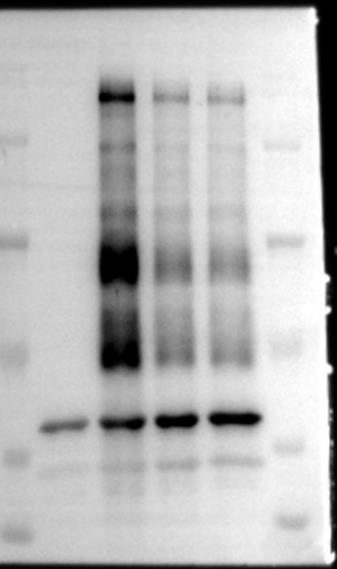

Supplement: Figure 5—figure supplement 2—source data 2. [file elife-98593-fig5-figsupp2-data2.zip › Figure 5–Figure Supplement 2–Source Data 2/Figure 5–Figure Supplement 2A-S.tif]

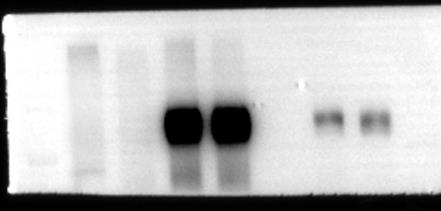

Supplement: Figure 5—figure supplement 2—source data 2. [file elife-98593-fig5-figsupp2-data2.zip › Figure 5–Figure Supplement 2–Source Data 2/Figure 5–Figure Supplement 2E-ACE2-V5.tif]

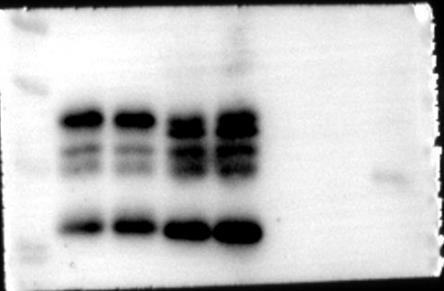

Supplement: Figure 5—figure supplement 2—source data 2. [file elife-98593-fig5-figsupp2-data2.zip › Figure 5–Figure Supplement 2–Source Data 2/Figure 5–Figure Supplement 2E-Myc-RhoA.tif]

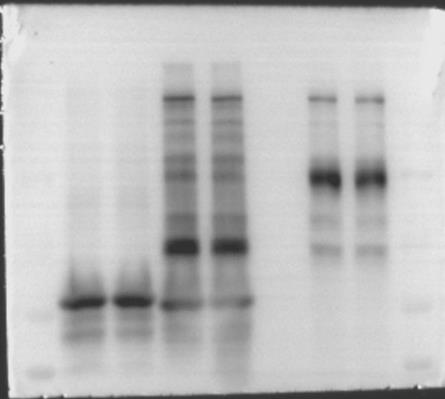

Supplement: Figure 5—figure supplement 2—source data 2. [file elife-98593-fig5-figsupp2-data2.zip › Figure 5–Figure Supplement 2–Source Data 2/Figure 5–Figure Supplement 2E-S.tif]

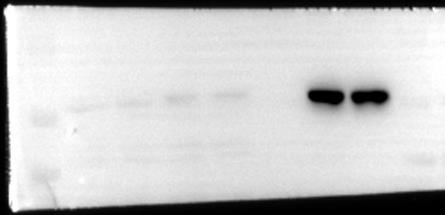

Supplement: Figure 5—figure supplement 2—source data 2. [file elife-98593-fig5-figsupp2-data2.zip › Figure 5–Figure Supplement 2–Source Data 2/Figure 5–Figure Supplement 2E-Tubulin.tif]

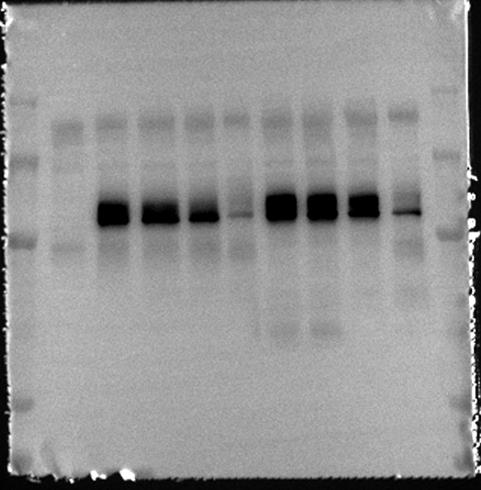

Supplement: Figure 5—figure supplement 3—source data 2. [file elife-98593-fig5-figsupp3-data2.zip › Figure 5–Figure Supplement 3–Source Data 2/Figure 5–Figure Supplement 3E-ACE2.tif]

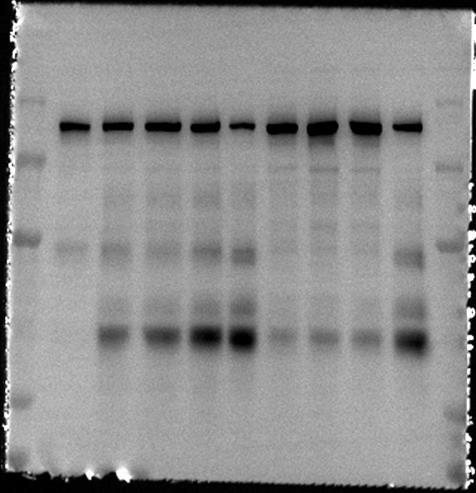

Supplement: Figure 5—figure supplement 3—source data 2. [file elife-98593-fig5-figsupp3-data2.zip › Figure 5–Figure Supplement 3–Source Data 2/Figure 5–Figure Supplement 3E-S.tif]

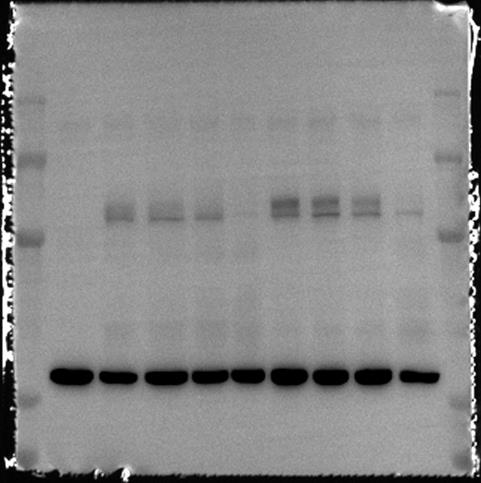

Supplement: Figure 5—figure supplement 3—source data 2. [file elife-98593-fig5-figsupp3-data2.zip › Figure 5–Figure Supplement 3–Source Data 2/Figure 5–Figure Supplement 3E-Tubulin.tif]

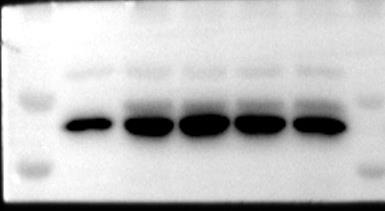

Supplement: Figure 5—figure supplement 3—source data 2. [file elife-98593-fig5-figsupp3-data2.zip › Figure 5–Figure Supplement 3–Source Data 2/Figure 5–Figure Supplement 3F-Actin.tif]

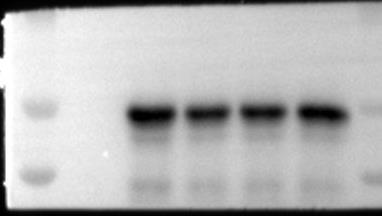

Supplement: Figure 5—figure supplement 3—source data 2. [file elife-98593-fig5-figsupp3-data2.zip › Figure 5–Figure Supplement 3–Source Data 2/Figure 5–Figure Supplement 3F-N.tif]

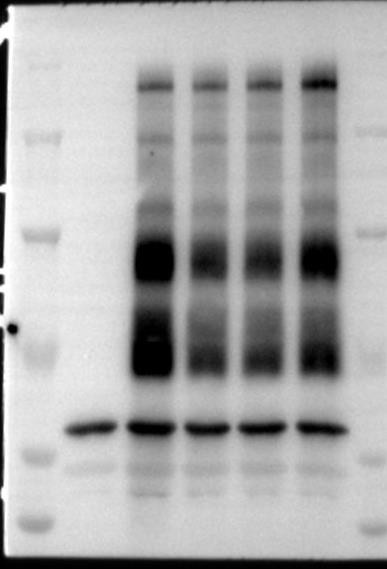

Supplement: Figure 5—figure supplement 3—source data 2. [file elife-98593-fig5-figsupp3-data2.zip › Figure 5–Figure Supplement 3–Source Data 2/Figure 5–Figure Supplement 3F-S.tif]

G

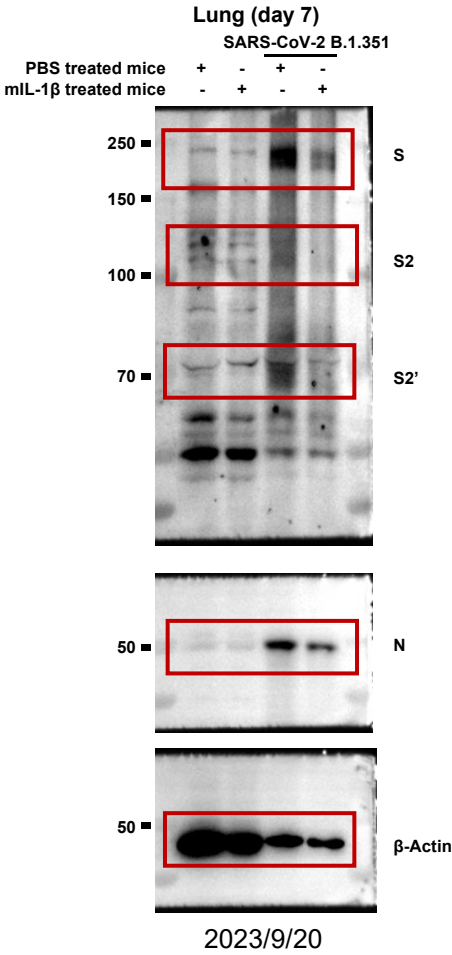

**Figure 6-Source Data 1.** Original membranes corresponding to Figure 6G.

Supplement: Figure 6—source data 1. [file elife-98593-fig6-data1.pdf]

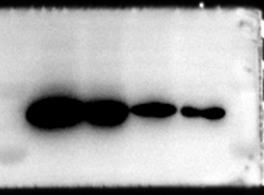

Supplement: Figure 6—source data 2. [file elife-98593-fig6-data2.zip › Figure 6 - Source data 2/Figure 6G-Actin.tif]

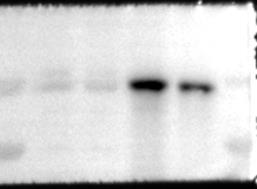

Supplement: Figure 6—source data 2. [file elife-98593-fig6-data2.zip › Figure 6 - Source data 2/Figure 6G-N.tif]

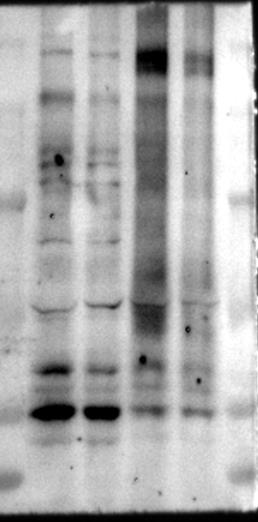

Supplement: Figure 6—source data 2. [file elife-98593-fig6-data2.zip › Figure 6 - Source data 2/Figure 6G-S.tif]

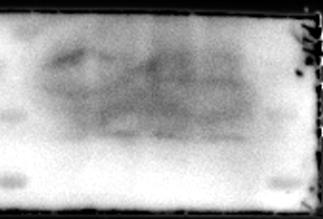

Supplement: Figure 6—figure supplement 2—source data 2. [file elife-98593-fig6-figsupp2-data2.zip › Figure 6–Figure Supplement 2–Source Data 2/Figure 6–Figure Supplement 2E-Actin.tif]

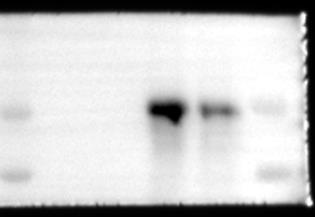

Supplement: Figure 6—figure supplement 2—source data 2. [file elife-98593-fig6-figsupp2-data2.zip › Figure 6–Figure Supplement 2–Source Data 2/Figure 6–Figure Supplement 2E-N.tif]

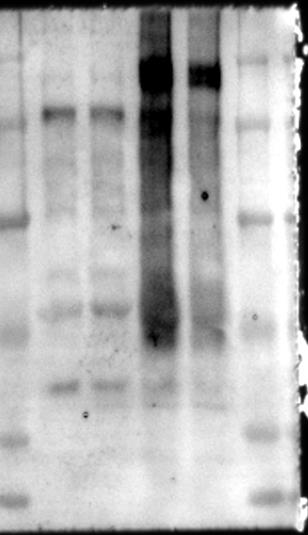

Supplement: Figure 6—figure supplement 2—source data 2. [file elife-98593-fig6-figsupp2-data2.zip › Figure 6–Figure Supplement 2–Source Data 2/Figure 6–Figure Supplement 2E-S.tif]

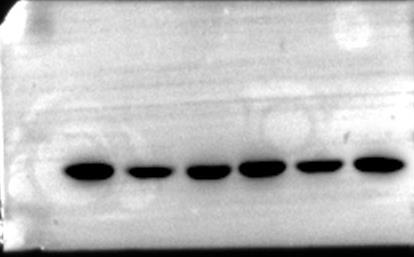

Supplement: Figure 7—source data 2. [file elife-98593-fig7-data2.zip › Figure 7 - Source data 2/Figure 7G-Actin.tif]

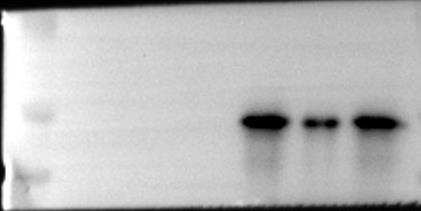

Supplement: Figure 7—source data 2. [file elife-98593-fig7-data2.zip › Figure 7 - Source data 2/Figure 7G-N.tif]

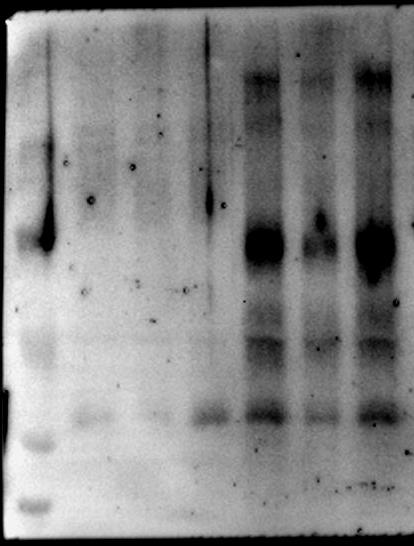

Supplement: Figure 7—source data 2. [file elife-98593-fig7-data2.zip › Figure 7 - Source data 2/Figure 7G-S.tif]

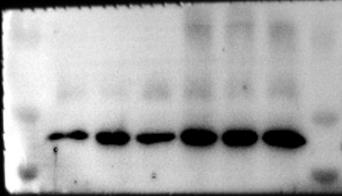

Supplement: Figure 7—figure supplement 2—source data 2. [file elife-98593-fig7-figsupp2-data2.zip › Figure 7–Figure Supplement 2–Source Data 2/Figure 7–Figure Supplement 2D-Actin.tif]

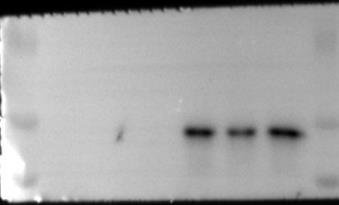

Supplement: Figure 7—figure supplement 2—source data 2. [file elife-98593-fig7-figsupp2-data2.zip › Figure 7–Figure Supplement 2–Source Data 2/Figure 7–Figure Supplement 2D-N.tif]

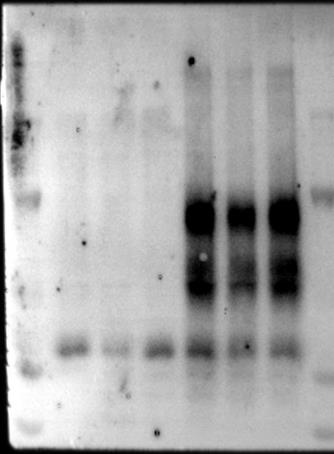

Supplement: Figure 7—figure supplement 2—source data 2. [file elife-98593-fig7-figsupp2-data2.zip › Figure 7–Figure Supplement 2–Source Data 2/Figure 7–Figure Supplement 2D-S.tif]
