# Supplementary material for: Interleukin-1 prevents SARS-CoV-2-induced membrane fusion to restrict viral transmission via induction of actin bundles
Source: eLife. 2025 Feb 12;13:RP98593. doi: 10.7554/eLife.98593 (PMC11820142; doi:10.7554/eLife.98593)
Supplement: Figure 7—source data 1. [file elife-98593-fig7-data1.pdf]

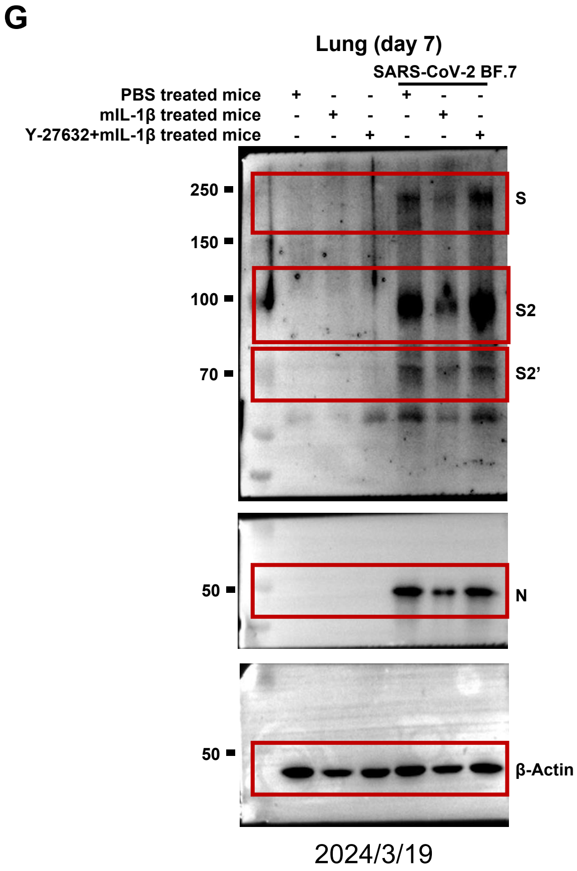

Figure 7-Source Data 1. Original membranes corresponding to Figure 7G.
